# Comparison of Meta-Analytical Estimates Between Surgical Repair and Transcatheter Edge-to-Edge Repair for Atrial Functional Mitral Regurgitation

**DOI:** 10.1093/icvts/ivaf269

**Published:** 2025-12-24

**Authors:** Sherif Khairallah, Mohamed Rahouma, Michelle Demetres, Leonardo Girardi, Mario Gaudino, Aina Hirofuji, Mark Reisman, Stephanie L Mick

**Affiliations:** Cardiothoracic Surgery Department, Weill Cornell Medicine (WCM), New York-Presbyterian Hospital, New York, NY 10065, United States; Surgical Oncology Department, National Cancer Institute, Cairo University, Cairo, 11796, Egypt; Cardiothoracic Surgery Department, Weill Cornell Medicine (WCM), New York-Presbyterian Hospital, New York, NY 10065, United States; Surgical Oncology Department, National Cancer Institute, Cairo University, Cairo, 11796, Egypt; Weill Cornell Medicine (WCM), Samuel J. Wood Library & C.V. Starr Biomedical Information Center, New York, NY, 10065, USA; Cardiothoracic Surgery Department, Weill Cornell Medicine (WCM), New York-Presbyterian Hospital, New York, NY 10065, United States; Cardiothoracic Surgery Department, Weill Cornell Medicine (WCM), New York-Presbyterian Hospital, New York, NY 10065, United States; Cardiothoracic Surgery Department, Weill Cornell Medicine (WCM), New York-Presbyterian Hospital, New York, NY 10065, United States; Structural Heart Program, Weill Cornell Medical Center, New York, NY, 10065, USA; Cardiothoracic Surgery Department, Weill Cornell Medicine (WCM), New York-Presbyterian Hospital, New York, NY 10065, United States

**Keywords:** AFMR, TEER, MVR, meta-analysis, Mitra-Clip

## Abstract

**OBJECTIVES:**

Atrial functional mitral regurgitation (MR) lacks well-defined treatment guidelines. Medical therapy alone is insufficient, and either TEER (transcatheter edge-to-edge) or surgery is recommended. Short- and long-term comparative outcomes remain unclear. We aim to address this gap using available data.

**METHODS:**

We performed a meta-analysis of studies examining the outcomes of surgery and/or transcatheter edge-to-edge. MEDLINE, EMBASE, and the Cochrane Library were assessed. The incidence rate of late severe MR was the primary outcome. A random model was performed. Leave-one-out, subgroup, and meta-regression analyses were conducted.

**RESULTS:**

Thirty-two studies (1923 patients); 20 in surgery (1166) vs 12 in TEER (757), were selected. TEER patients were, on average, 10 years older, with twice the rate of New York Heart Association Classification (NYHA) III/IV symptoms and more than double the Society of Thoracic Surgeons Risk (STS) score. At a weighted mean follow-up of 3.2 years, compared to isolated transcatheter, surgery was associated with decreased incidence of late severe MR (2.53 vs 6.66 events per 100 person-years, *P*-interaction = .03), late all-cause mortality (3.00 vs 8.84, *P*-interaction = .024), late heart failure hospitalization (4.44 vs 17.03, *P*-interaction < .01), and late NYHA III/IV (2.98 vs 22.47, *P*-interaction < .01). However, significantly better long-term outcomes associated with surgery showed high heterogeneity. There were no differences in early all-cause mortality, early cardiac-specific mortality, late cardiac-specific mortality, postprocedural morbidities, or atrial diameter. On meta-regression, preprocedural heart failure (β = 0.0224, *P* < .01) and coronary artery disease (β = 0.0294, *P* < .00001) were linked to increased late severe MR. Older age, hypertension, mitral valve replacement, and associated aortic valve surgery were linked to increased late all-cause mortality.

**CONCLUSIONS:**

Surgery and isolated transcatheter edge-to-edge repair showed comparable short-term outcomes, with surgery appearing more effective long-term; however, due to study limitations and heterogeneity, these findings are hypothesis-generating and require validation through prospective studies.

**CLINICAL REGISTRATION NUMBER:**

PROSPERO website: CRD42024504022.

## GRAPHICAL ABSTRACT

**Figure:**
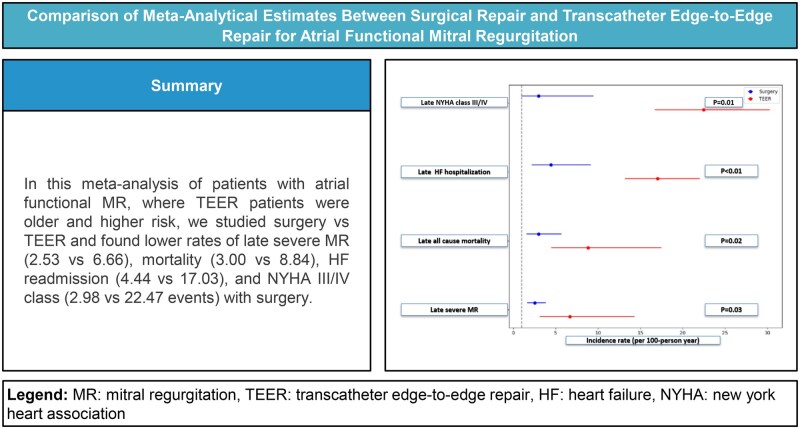

## FUTURE PERSPECTIVE

While we hypothesize that the superior outcomes of surgery compared to isolated TEER is related to the direct repair of the dilated mitral annulus, concomitant tricuspid valve repair and atrial fibrillation ablation, the causal relationship remains unclear. Randomized controlled trials are needed to further address this issue and establish optimal treatment recommendations for atrial functional mitral regurgitation.

## INTRODUCTION

Functional mitral regurgitation (FMR) is classified into 2 subtypes: atrial functional mitral regurgitation (AFMR) and ventricular functional mitral regurgitation (VFMR), each with distinct pathophysiological mechanisms.^1–3^ VFMR typically results from ischaemic cardiomyopathy, characterized by reduced ejection fraction (EF), left ventricular dilation leading to papillary muscle displacement, and mitral annular dilation. AFMR, on the other hand, is often a long-term consequence of atrial fibrillation (AF), typically with preserved EF and progressive mitral annular dilation due to left atrial remodelling.^3–5^ AFMR was first described in 2010 by Gertz *et al.* Additionally, there is no consensus regarding the accurate definitions^6^ and specific echocardiographic parameters for AFMR.^5^ Some definitions focus on the presence of AF with progressive left atrial dilation, while others consider a history of AF exceeding 1 or 3 years. EF cutoffs also differ, ranging between 45% and 55%. Alternatively, some define AFMR as progressive left atrial dilation with preserved EF in patients with heart failure (HF), regardless of heart rhythm.^6^ Given the increasing prevalence of heart failure with preserved ejection fraction and AF over the past 50 years,^7^ AFMR is anticipated to become a more common form of FMR in the future.^8^

Treatment strategies differ. For VFMR, the primary approach combines medical management of HF with surgical mitral annulus reduction or transcatheter edge-to-edge repair (TEER).^9^^,^^10^ For AFMR, there is no consensus on the optimal treatment strategy (surgery vs TEER). Recent guidelines suggest surgery as a class IIb recommendation following failure of medical treatment, but there is still no consensus on utilizing TEER in AFMR.^11–13^ This meta-analysis aims to consolidate the evidence, with preliminary evaluation and comparison of surgical intervention vs TEER for AFMR using currently available data.

## METHODS

This study was performed following the Preferred Reporting Items for Systematic Reviews and Meta-Analyses (PRISMA).^14^ PROSPERO registration number is: CRD42024504022.

### Search strategy

A medical librarian performed comprehensive searches to identify studies that compared or adopted surgical repairs and/or TEER for AFMR.

Searches were run on 6 December 2023, in the following databases: Ovid MEDLINE (1946 to Present), Ovid EMBASE (1974 to present), and The Cochrane Library (Wiley). Search strategy included all appropriate controlled vocabulary for the concepts of “atrial fibrillation” and “functional mitral regurgitation”. Full search strategies for all databases are available in **Table S1**. To limit publication bias, there were no restrictions on language, publication date, or article type in the search strategy.

### Study selection

Retrieved studies were screened for inclusion. Titles and abstracts were reviewed against predefined inclusion/exclusion criteria by 2 independent reviewers. Discrepancies were resolved by consensus. For final inclusion, the full text was then retrieved and screened by 2 independent reviewers (S.K. and S.M.). No automation tools used for this process. Inclusion and exclusion criteria of studies are provided in **Table S2**.

### Clinical outcomes and definitions

The primary outcome was the incidence rate (IR) of late severe MR (3+, and 4+). Secondary outcomes were early (30-day) stroke, early acute kidney injury (AKI), early all-cause mortality, early cardiac-specific mortality, early HF hospitalization, early reoperation/reintervention, postprocedural atrial, and ventricular reverse remodelling (left atrial diameter [mm], mean left ventricular end-systolic diameter (LVESD) [mm], mean left ventricular end-diastolic diameter (LVEDD) [mm], mean EF, late (beyond 30-day follow-up) reoperation, late all-cause mortality, late cardiac-specific mortality, late postprocedural New York Heart Association Classification (NYHA) III/IV, late stroke, late HF hospitalization/readmission). Data extracted from included studies were study details, baseline patient data such as demographics and comorbidities/history, echocardiographic data, procedural details, and outcomes such as postprocedural MR and all-cause mortality; a complete list of extracted data variables are given in **Table S3**.

### Risk of bias (quality) assessment

Retrospective studies were assessed using the Newcastle-Ottawa scale (NOS).^15^ Using NOS, each study was judged on a total of 8 quality items from 3 categories: selection of study groups, comparability of groups, and the ascertainment of either the exposure or outcome of interest for case-control or cohort studies, respectively. Stars added for each quality item serve as a quick visual assessment with the highest quality studies receiving a maximum of 9 stars (**Table S4**). Two independent reviewers (S.K., M.R.) performed the quality assessment, and disagreements were resolved by a senior reviewer (S.M.). Publication bias was assessed visually using a funnel plot and statistically using Egger’s test.

### Statistical analysis

Continuous variables were expressed as mean (SD). Short-term categorical variables were presented as proportions (pooled events), and time-to-event variables were presented as IR, with 95% CI. The minimum number of studies required to generate meta-analytic estimates was 3. All analyses were performed using “metafor”^16^ and “meta”^17^ packages in the R program (version 4.1.1) within RStudio.

For long-term events that were reported using Kaplan-Meier curves, the curves were digitized, and the individual patients’ data were reconstructed using an iterative algorithm^18^ that was applied to solve the Kaplan-Meier equations originally used to produce the published graphs. This algorithm used digitized Kaplan–-Meier curve data obtained by the Graph Grabber software package (Quintessa, Oxfordshire, UK) to find numerical solutions to the inverted Kaplan-Meier equation. To account for different follow-up times in the studies, an underlying Poisson process with a constant event rate was assumed with a total number of events observed within a group of the total person-time of follow-up for that group calculated from study follow-up.

Study heterogeneity was assessed using the Cochran Q statistic and the *I*^2^ test. For the primary outcome as well as for late all-cause mortality, a leave-one-out sensitivity analysis and funnel plots were performed for each of the study groups and for the whole cohort.

### Analysis of subgroups or subsets

Subgroup analysis was used to compare surgery vs TEER for primary and secondary outcomes. There were no studies directly comparing both approaches; hence, we used a meta-analytical approach where a test for subgroup differences that checks whether the random effects estimate in the 2 subgroups differ was applied to the estimate for each reported cohort. This was done via metagen() or rma.uni() to calculate the Q statistic and *P*-value in meta or metafor packages, respectively^16^^,^^17^; This approach has been successfully utilized in prior meta-analyses.^19–21^ Meta-regression was used to assess the effect of age, female%, preprocedural factors (EF, NYHA class III/IV, hypertension, diabetes, chronic kidney disease, coronary artery disease [CAD], chronic obstructive pulmonary disease, dyslipidaemia, smoking, concomitant AF, EuroSCORE II and Society of Thoracic Surgeons Risk (STS)-PROM score means, left atrial diameter [mm], left atrial volume [mL], left atrial volume index [mL/m^2^], LVEDD [mm], LVEDV [mL], LVESD [mm], effective regurgitation orifice area [cm^2^], MR volume [mL], and concomitant ≥2+ TR), publication year, mitral valve repair %, mitral valve replacement (MVR)%, associated coronary artery bypass grafting%, associated aortic valve (AV) procedures% on the IR of the primary outcome (for the entire cohort, and for each study group separately). A random-effect model (inverse variance method) was used.

## RESULTS

### Characteristics of eligible studies

A total of 5516 articles were retrieved, and deduplication yielded 3877 articles. Thirty-two studies were ultimately included; the PRISMA flowchart is shown in **Figure S1**. A total of 1923 patients were included in this analysis (1166 in surgery vs 757 in TEER). The weighted mean follow-up time was 3.26 years (4.48 in surgery vs 1.41 in TEER). The weighted mean age was higher in the TEER group (69.52 [4.68] years in surgery vs 79.44 [1.4] years in TEER). The proportion of female patients was greater in the TEER group (55.33 [4.96%]) vs surgery (48.32 [14.22%]). The weighted mean preprocedural NYHA class III/IV was higher in the TEER group (81.96 ([10.66]) vs surgery (41.4 [12.9]). No significant difference was observed in the weighted mean preoperative EF (59.68 [15.6%] in surgery vs 57.98 [6.74%] in TEER). Systemic hypertension was more prevalent in the TEER group, with a weighted mean% of 79.08 (4.22%) vs 53.02 (20.7%) in surgery. The weighted mean of the preprocedural EuroSCORE II and STS-PROM scores were lower in surgery compared with TEER (EuroSCORE II: 3.87 [1.75] vs 5.05 [1.57], STS-PROM: 2.48 [3.26] vs 6.46 [1.65] for surgical and TEER groups, respectively). MR severity, indicated by weighted mean preprocedural left atrial and MR volumes, was higher in surgery (left atrial volume: 137.58 [36.48 mL]; MR volume: 58.05 [2.41 mL]) than TEER (left atrial volume: 77.1 [17.27 mL]; MR volume: 46.6 [5.57 mL]). The weighted mean% of preprocedural concomitant TR ≥ 2+ was higher in surgery (71.58 [12.5%]) than in TEER (56.19 [17.22%]). Baseline characteristics of the entire included cohort, the preprocedural echocardiographic data, as well as the weighted mean and weighted mean% of different variables are reported in **Table 1**, and **Tables S5 and S6**. No patients in the TEER group underwent transcatheter tricuspid valve (TV) repair or AF ablation procedures (**Table S7**). Detailed AF ablation and TV repair procedures can be found in **Table S8**.

**Table 1.** Preprocedural Demographic Characteristics of Patients in the Included Studies

Study	Arm	Study years	Study type	Patients no	Age (years) (mean/median)	Female %	Preprocedural EF	Hypertension %	Diabetes %	CKD %	CAD %	COPD %	Dyslipidaemia %	Smoking %	Preprocedural NYHA III and IV	Preprocedural concomitant A Fib %	Preprocedural EuroSCORE II mean/median	Preprocedural STS risk score mean/median	Follow-up (months) (mean/median)
Hirji et 2020	S	2002-2019	R	94	67.6	70.2	60	63.8	70.2	6.4	0	5.3	51.1	NA	42.6	37.2	NA	2.48	84
Takahashi 2020	S	2008-2016	R	45	72	46.7	63	36	46.7	16	4	11	7	NA	67	100	NA	NA	31
Deferm 2021	S	2010-2018	R	97	73	68	55	87.6	68	2.1	12.4	NA	67	NA	54	76.3	4.2	NA	39.6
Balogh 2020	S	2003-2017	R	131	71.99	21.4	59.05	60.5	0.8	0.8	0	10	NA	NA	71	93.1	NA	NA	63.6
Sakagushi 2019	S	NA	R	20	68	55	63.3	60	25	25	NA	NA	30	NA	35	100	3.4	NA	28
Morisaki 2022	S	2008-2021	R	31	72.5	25	PA: 60, MVR: 63	56.3	6.3	6.3	NA	12.5	12.5	50	56.3	100	2.81	NA	20.4
Ohba Masanao 2020	S	NA	R	3	69.6	0	66.6	NA	NA	NA	NA	NA	NA	NA	0	100	NA	NA	NA
Vohra 2012	S	2007-2011	R	20	77.5	70	NA	65	10	10	10	NA	65	30	50	100	8.1	NA	18
Kaneyuki 2020	S	2011-2018	R	40	69	32	NA	NA	NA	NA	NA	NA	NA	NA	7.5	100	NA	NA	42
Chen 2020	S	2008-2018	R	82	63.6	52.2	61.3	32.9	7.3	7.3	NA	4.9	NA	11	72	100	NA	NA	26.1
Matsumori 2020	S	2000-2019	R	22	73.5	31.9	63.8	18.1	27.2	27.2	NA	63.6	27.2	36.3	31.7	100	NA	NA	42.96
Tanaka 2020	S	2015-2018	R	10	72.4	60	52.2	60	10	10	NA	NA	NA	NA	NA	60	NA	NA	20.36
Carino 2021	S	NA	R	20	72.2	90	55	55	20	20	5	10	15	NA	40	100	3.8	NA	18
Kim 2023	S	2000-2020	R	36	66.5	53	57.9	39	23	23	0	NA	NA	NA	27	100	NA	NA	50.7
Mesi 2021	S	2010-2018	R	**23**	70.4	56.4	60	94.9	46.2	46.2	48.7	NA	74.4	53.8	51.3	77	NA	NA	22
Kihara 2009	S	2000- 2004	R	12	69	50	63	NA	NA	NA	0	NA	NA	NA	0	100	NA	NA	12
Kawamoto 2022	S	2001-2019	R	50	74	42	69	66	4	4	NA	54	24	NA	NA	82	NA	NA	55.2
Ye 2023^66^	S	2010 - 2019	P	247	62.6	50.7	51	21.4	27.1	27.1	22.5	14.9	NA	42.6	65	100	2.3	NA	63.6
Fan 2023	S	2012- 2015	R	60	63	36.7	56	26.7	16.7	16.7	18.3	6.7	NA	NA	33.3	100	2.5	NA	95.76
Wagner 2022	S	2000-2020	R	123	62	55.3	58	58		NA	0	NA	NA	NA	NA	60.2	NA	NA	33.3
Tanaka 2022	I (E-E)	2010-2021	R	118	80	60.2	59.7	75.4	28.8	28.8	69.5	17.8	NA	NA	89	90.7	3.4	NA	12
Doldi 2022	I (E-E)	2008-2019	R	126	80.3	61.1	57.1	84.9	17	17	41.4	16.7	NA	NA	85.7	78.6	4.7	NA	24.2
Yoon 2022	I (E-E)	2007- 2020	R	116	78.8	56	61.9	83.6	30.2	30.2	42.2	7.8	NA	NA	94.8	72.4	NA	8.5	18
Benito‐González 2021	I (E-E)	2012-2021	R	48	78	52.1	58	75	35.4	35.4	56.3	29.2	50	NA	89.6	100	4	4.6	12
Claeys 2021	I (E-E)	2011-2019	R	52	79	54	59	79	21	21	30	NA	NA	NA	71	62	8	NA	15
Li 2022	I (E-E)	6/2021-11/2021	P	5	70	60	58	60	0	0	0	NA	NA	NA	80	100	NA	5.14	3
Simard 2022	I (E-E)	2014-2020	R	21	82	23.8	60	81	19	19	85.7	19	NA	NA	90.5	76.2	NA	8.4	12
Sodhi 2022	I (E-E)	2018-2019	P	53	79.4	60.3	60.06	NA	26.4	26.4	NA	17	NA	NA	48.9	100	NA	6.8	12
Masiero 2023	I (E-E)	2016-2020	R	71	79	58	55	85	26	26	34	NA	46	18	73	76	4.2	NA	15.9
Rottländer 2022	I (E-E)	2014-2020	R	20	82.8	65	54	85	30	30	55	NA	NA	0	80	80	NA	NA	18
Yoshida 2021	I (E-E)	2010-2018	R	40	83	52.5	58	78	33	33	27	30	30	NA	98	100	NA	7.8	10.9
Popolo 2021	I (E-E)	2009- 2021	R	84	81	61	55	83	22	22	NA	21	21	NA	83	100	6	4	15.16

Abbreviations: A Fib, atrial fibrillation; CAD, coronary artery disease; CKD, chronic kidney disease; COPD, chronic obstructive pulmonary disease; EF, ejection fraction; EuroSCORE, European System for Cardiac Operative Risk Evaluation; I (E-E), intervention (edge to Edge); MVR, mitral valve replacement; NA, not avialable; NYHA, New York Heart Association; P, prospective; PA, patch augmentation; R, retrospective study; S, Surgery group; STS Score, Society of Thoracic Surgeons Risk Score.

### Meta-analysis

#### Primary outcome: incidence rate of late severe mitral regurgitation (3+ and 4+)

**Figure 1.**
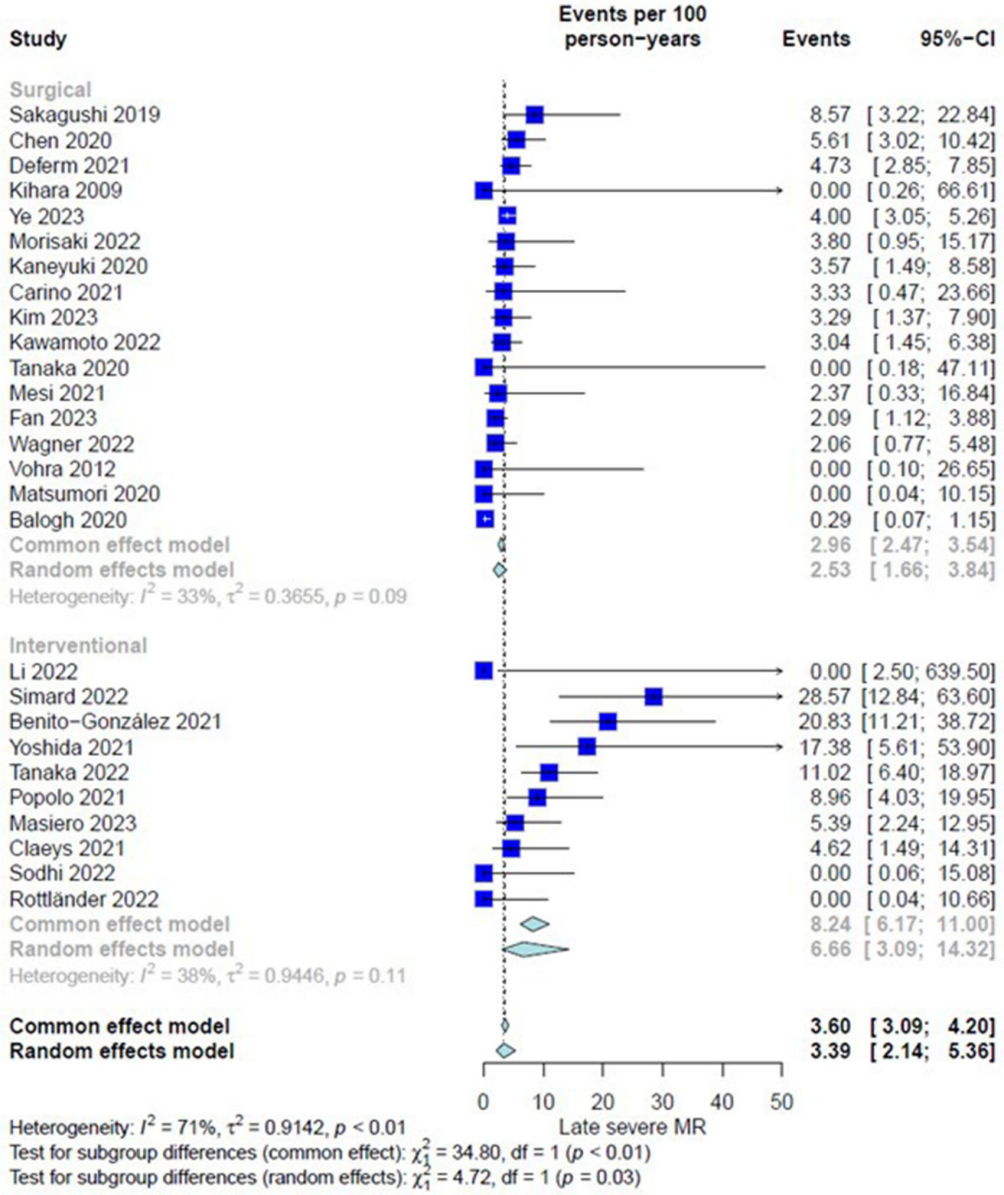
Forest Plot, Subgroup Differences Between Surgery Group and Transcatheter Edge To Edge Repair Group for Incidence Rate of Late (Follow-Up) Recurrent Severe Mitral Regurgitation (MR).

Twenty-seven studies (1478 patients) were assessed. Overall IR of late severe MR for the entire cohort was 3.39 event per 100 person-year (PY) (CI = 2.14-5.36). There was high heterogeneity among included studies (*I*^2^ = 71%, *P *< .01). Subgroup analysis based on the repair intervention showed IR of 2.53 event per 100 PY (CI = 1.66-3.84) and 6.66 event per 100 PY (CI = 3.09-14.32) in Surgery and TEER groups, respectively (interaction-*P *= .03) (**Figure 1**, **Table 2**). Leave-one-out analysis showed robustness of the obtained estimate. Funnel plot (**Figure S2**) demonstrates absence of publication bias in both groups (Egger intercept was −0.786 [0.492], *P *= .131 vs −1.467 [1.0693], *P *= .207 in surgery and TEER groups, respectively).

**Table 2.** Outcomes Summary

Outcome	Studies	Patients	Estimate % (95% CI)	Heterogeneity (*I*^2^, *P*-value)	*P*-interaction
Early severe MR	29	1580	4.78 (3.02-7.49)	66%, *P* < .01	**<.01**
Surgical	17	829	2.54 (1.46-4.39)	0%, *P* = .66	
Interventional	12	751	8.86 (5.42-14.17)	71%, *P* < .01	
Early stroke	16	1031	2.62 (1.72-3.98)	0%, *P* = .72	.68
Surgical	13	921	2.49 (1.59-3.88)	0%, *P* = .87	
Interventional	3	110	3.74 (0.56-21.04)	53%, *P* = .12	
Early AKI	13	650	4.09 (2.38-6.94)	17%, *P* = .27	.0504
Surgical	11	558	3.20 (1.73-5.85)	8%, *P* = .37	
Interventional	2	92	8.06 (3.99-15.61)	0%, *P* = .98	
Early all-cause mortality	30	1860	2.37 (1.68-3.34)	0%, *P* = .84	.0515
Surgical	19	1143	1.65 ((1.00-2.72)	0%, *P* = .94	
Interventional	11	717	3.27 (2.04-5.21)	0%, *P* = .62	
Early cardiac-specific mortality	26	1418	1.92 (1.22-3.02)	0%, *P* = .95	.33
Surgical	16	749	1.49 (0.74-2.95)	0%, *P* = .96	
Interventional	10	669	2.34 (1.28-4.24)	0%, *P* = .66	
Early HF hospitalization	18	940	3.33 (1.83-5.99)	44%, *P* = .02	.96
Surgical	15	784	3.19 (1.58-6.34)	47%, *P* = .02	
Interventional	3	156	3.28 (1.31-8.00)	0%, *P* = .54	
Early reoperation/reintervention	20	969	2.96 (1.85-4.69)	2%, *P* = .44	.22
Surgical	15	721	3.26 (1.89-5.56)	9%, *P* = .35	
Interventional	5	248	1.50 (0.48-4.57)	0%, *P* = .71	
Postoperative mean LVESD (mm)	10	705	32.93 (30.71-35.15)	89%, *P* < .01	.30
Surgical	7	544	33.94 (32.36-35.52)	80%, *P* < .01	
Interventional	3	161	30.42 (24.01-36.83)	95%, *P* < .01	
Postoperative mean LVEDD (mm)	13	776	51.69 (46.57-56.81)	94%-*P* < .01	.33
Surgical	9	517	49.18 (47.48-50.88)	89%, *P* < .01	
Interventional	4	259	57.78 (40.43-75.12)	98%, *P* < .01	
Postprocedural Lt atrial diameter (mm)	10	555	51.10 (48.91-53.28)	87%, *P* < .01	.34
Surgical	7	345	50.45 (48.11-52.79)	86%, *P* < .01	
Interventional	2	210	56.15 (44.64-67.66)	87%, *P* < .01	
Postprocedural mean EF	15	1014	57.27 (55.38-59.15)	95%, *P* < .01	.79
Surgical	9	643	57.03 (54.80-59.26)	94%, *P* < .01	
Interventional	6	371	57.60 (54.07-61.13)	97%, *P* < .01	
Late severe MR (3+ and 4+)	27	1478	3.39 (2.14-5.36)	71%, *P* < .01	**.03**
Surgical	17	1021	2.53 (1.66-3.84)	33%, *P* = .09	
Interventional	10	457	6.66 (3.09-14.32)	38%, *P* = .11	
Late reoperation	17	690	1.62 (1.03-2.55)	0%, *P* = .52	.17
Surgical	12	525	1.45 (0.87-2.41)	0%, *P* = .60	
Interventional	5	165	3.12 (1.18-8.24)	0%, *P* = .82	
Late all-cause mortality	25	1617	4.63 (2.72-7.87)	58%, *P* < .01	**<.01**
Surgical	14	924	3.00 (1.57-5.72)	88%, *P* < .01	
Interventional	11	693	8.84 (4.47-17.47)	58%, *P* < .01	
Late cardiac-specific mortality	16	859	1.36 (0.54-3.45)	80%, *P* < .01	.23
Surgical	11	552	1.06 (0.47-2.40)	51%, *P* = .03	
Interventional	5	307	3.33 (0.61-18.09)	40%, *P* = .16	
Late stroke	13	884	0.51 (0.32-0.83)	64%, *P* < .01	.35
Surgical	10	774	0.47 (0.28-0.78)	18%, *P* = .27	
Interventional	3	110	1.99 (0.10-39.25)	74%, *P* = .02	
Late HF hospitalization/readmission	16	968	7.15 (4.17-12.26)	87%, *P* < .01	**<.01**
Surgical	10	666	4.44(2.16-9.14)	89%, *P* < .01	
Interventional	6	302	17.03(13.17-22.03)	0%, *P* = .75	
Late posttreatment NYHA III/IV	13	587	15.56 (9.48-25.35)	96%, *P* < .01	**<.01**
Surgical	3	114	33.95 (24.90-46.28)	51%, *P* = .13	
Interventional	10	473	43.80 (24.59-78.01)	97%, *P* < .01	

Abbreviations: AKI, acute kidney injury; EF, ejection fraction; HF, heart failure; I², heterogeneity index; Lt, left; LVEDD, left ventricular end-diastolic diameter; LVESD, left ventricular end-systolic diameter; MR, mitral regurgitation; NYHA, New York Heart Association. Bold Values: Statistically significant values.

On meta-regression, there was a significant association between increased IR of late severe MR and preprocedural NYHA class III/IV (β = 0.0224 [0.0088], *P *= .0111), and preprocedural CAD (β = 0.0294 [0.0076], *P *< .00001) across the entire group. In the surgery group, female gender was associated with increased IR of late severe MR (β = 0.0262 [0.0099], *P *= .0084). In the TEER group, preprocedural NYHA class III/IV (β = 0.0812 [0.0217], *P *< .001) and mean MR volume (β = 0.1085 [0.0486], *P *= .0256) were associated with increased IR of late severe MR, while female gender (β = −0.055 [0.023], *P *= .0167) was associated with decreased IR of late severe MR (**Table 3**).

**Table 3.** Meta-Regression for the (i) Primary Outcome Among the Entire Group, Surgery Group, and Intervention and (ii) Late All-Cause Mortality Among the Entire Group, Surgery Group, and Intervention

	Entire group	Surgery group	Intervention
**Variables**			
	(β^a^ ± SD, *P*-value)	(β^a^ ± SD, *P*-value)	(β^a^ ± SD, *P*-value)
Late severe MR			
Age	0.0455 ± 0.0306, *P* = .1369	−0.0554 ± 0.0456, *P* = .2245	0.0266 ± 0.1881, *P* = .8878
Female %	0.0058 ± 0.0151, *P* = .6997	**0.0262 ± 0.0099, *P* = .0084**	−**0.055 ± 0.023, *P* = .0167**
Preprocedural EF	−0.0048 ± 0.0599, *P* = .9361	−0.0102 ± 0.0463, *P* = .8263	0.1833 ± 0.1559, *P* = .2396
Preprocedural HF (NYHA class III/IV)	0.0224 ± 0.0088, *P* = .0111	−0.0017 ± 0.0121, *P* = .8911	**0.0812 ± 0.0217, *P* < .001**
Hypertension	0.016 ± 0.01, *P* = .1076	0.0006 ± 0.0101, *P* = .9559	−0.0876 ± 0.0486, *P* = .0712
Diabetes	0.0211 ± 0.0157, *P* = .1785	0.0153 ± 0.0112, *P* = .173	0.0168 ± 0.0624, *P* = .7878
CKD	0.0299 ± 0.02, *P* = .1345	0.0104 ± 0.0199, *P* = .6009	0.0168 ± 0.0624, *P* = .7878
CAD	0.0294 ± 0.0076, *P* = 1e−04	0.0222 ± 0.0198, *P* = .2606	0.0207 ± 0.0156, *P* = .1853
COPD	−0.0064 ± 0.0232, *P* = .781	−0.0116 ± 0.0183, *P* = .5263	0.0725 ± 0.0476, *P* = .1274
Dyslipidaemia	−9e−04 ± 0.0132, *P* = .9436	0.0055 ± 0.0081, *P* = .4971	0.0089 ± 0.0233, *P* = .7020
Smoking	7e−04 ± 0.0092, *P* = .9396	−0.0095 ± 0.011, *P* = .3861	———
Publication year	0.1796 ± 0.1462, *P* = .2191	0.1287 ± 0.1238, *P* = .2986	−0.5031 ± 0.5210, *P* = .3343
Preprocedural concomitant AF	0.0034 ± 0.0169, *P* = .8421	0.0064 ± 0.0161, *P* = .6921	0.0138 ± 0.0271, *P* = .6112
Procedural EuroSCORE II mean	−0.0014 ± 0.1258, *P* = .9912	0.0005 ± 0.1335, *P* = .997	−0.186 ± 0.1356, *P* = .1701
Preprocedural STS risk score mean	0.0751 ± 0.2382, *P* = .7526	NA	0.0751 ± 0.2382, *P* = .7526
Preprocedural Lt atrial diameter (mm)	0.0189 ± 0.0317, *P* = .5499	0.0341 ± 0.0583, *P* = .5589	−0.0086 ± 0.0404, *P* = .8307
Preprocedural Lt atrial volume (mL)	−0.0085 ± 0.0066, *P* = .2018	0.0022 ± 0.0057, *P* = .6974	0.0167 ± 0.0092, *P* = .071
Preprocedural Lt atrial volume index (mL/m^2^)	0.0035 ± 0.0088, *P* = .6933	0.0011 ± 0.0071, *P* = .8753	0.0689 ± 0.0536, *P* = .1984
Group surgical LVEDD (mm)	0.0136 ± 0.0255, *P* = .5954	0.0091 ± 0.0585, *P* = .8764	0.021 ± 0.0248, *P* = .3967
Group surgical LVEDV (mL)	−0.0036 ± 0.0082, *P* = .6648	−0.0009 ± 0.005, *P* = .8516	−0.007 ± 0.0249, *P* = .7793
Group surgical LVESD (mm)	−0.0496 ± 0.0471, *P* = .293	−0.0608 ± 0.0372, *P* = .1022	0.0195 ± 0.0512, *P* = .7037
Preprocedural EROA (cm^2^)	0.1464 ± 5.1177, *P* = .9772	27.7745 ± 16.9037, *P* = .1004	11.4679 ± 7.1088, *P* = .1067
Preprocedural MR volume ml mean	−0.0004 ± 0.1687, *P* = .9979	NA	0.1085 ± 0.0486, *P* = .0256
Preprocedural concomitant ≥2+ TR	0.0013 ± 0.0109, *P* = .9048	0.003 ± 0.0095, *P* = .7495	0.0214 ± 0.0142, *P* = .132
MVr %	NA	0.0004 ± 0.0114, *P* = .9709	NA
MVR %	NA	−0.0156 ± 0.0229, *P* = .4949	NA
Group surgical tricuspid	NA	0.0167 ± 0.0126, *P* = .1835	NA
Associated CABG %	NA	0.0153 ± 0.012, *P* = .2027	NA
Associated aortic valve %	NA	0.0082 ± 0.013, *P* = .5295	NA
**Late all-cause mortality**			
Age	0.0750 ± 0.0338, *P* = .0263	0.0496 ± 0.067, *P* = .4592	−0.0711 ± 0.1577, *P* = .6522
Female %	−0.0011 ± 0.01790, *P* = .9500	−0.0067 ± 0.02, *P* = .7361	−0.0179 ± 0.0322, *P* = .5788
Preprocedural EF	0.0669 ± 0.0842, *P* = .4274	0.12 ± 0.0858, *P* = .1617	−0.0078 ± 0.1333, *P* = .9533
Preprocedural HF (NYHA class III/IV)	0.0171 ± 0.0110, *P* = .1209	0.0047 ± 0.0174, *P* = .7875	−0.0085 ± 0.0245, *P* = .7291
Hypertension	**0.0365 ± 0.0087, *P* < .001**	**0.0343 ± 0.009, *P* < .001**	**0.2034 ± 0.1057, P = 0.0542**
Diabetes	0.0187 ± 0.0200, *P* = .348	0.0238 ± 0.0179, *P* = .1833	−0.0483 ± 0.0484, *P* = .3176
CKD	0.0338 ± 0.025, *P* = .1764	0.0312 ± 0.0253, *P* = .2187	−0.0483 ± 0.0484, *P* = .3176
CAD	0.0155 ± 0.0148, *P* = .2958	0.0403 ± 0.0316, *P* = .2019	−0.0201 ± 0.0191, *P* = .2916
COPD	0.0130 ± 0.0268, *P* = .6272	0.0144 ± 0.0174, *P* = .4101	−0.0236 ± 0.0634, *P* = .7095
Dyslipidaemia	0.0093 ± 0.0165, *P* = .5713	0.0228 ± 0.0157, *P* = .1461	−0.0219 ± 0.013, *P* = .0908
Smoking	0.0272 ± 0.0267, *P* = .3092	0.0507 ± 0.0334, *P* = .1286	NA
Publication year	0.1781 ± 0.1519, *P* = .2410	0.0951 ± 0.1473, *P* = .5188	−0.0767 ± 0.5434, *P* = .8878
MVr %	NA	−**0.0342 ± 0.0108, *P* = .0015**	NA
MVR %	NA	**0.0508 ± 0.0131, *P* < .001**	NA
Group surgical tricuspid	NA	−0.0205 ± 0.0164, *P* = .2102	NA
Associated CABG %	NA	0.0508 ± 0.0464, *P* = .2738	NA
Associated aortic valve %	NA	0.0773 ± 0.0371, *P* = .0371	NA

a Negative beta value reflects an inversely proportional relationship between the outcome of interest and the covariate, while a positive beta value reflects a proportional relationship (eg, more MVR percentages were associated with higher late all-cause mortality).

Abbreviations: AF, atrial fibrillation; CABG, coronary artery bypass grafting; CAD, coronary artery disease; CKD, chronic kidney disease; COPD, chronic obstructive pulmonary disease; EF, ejection fraction; EROA, effective regurgitation orifice area; HF, heart failure; Lt, left; LVEDD, left ventricular end-diastolic diameter; LVEDV, left ventricular end-diastolic volume; LVESD, left ventricular end-systolic diameter; MR, mitral regurgitation; MVr, mitral valve repair; MVR, mitral valve replacement; NA, not available; NYHA, New York Heart Association; STS, Society of Thoracic Surgeons; TR, tricuspid regurgitation. Bold Values: Statistically significant values.

#### Secondary outcomes

##### Late all-cause mortality

**Figure 2.**
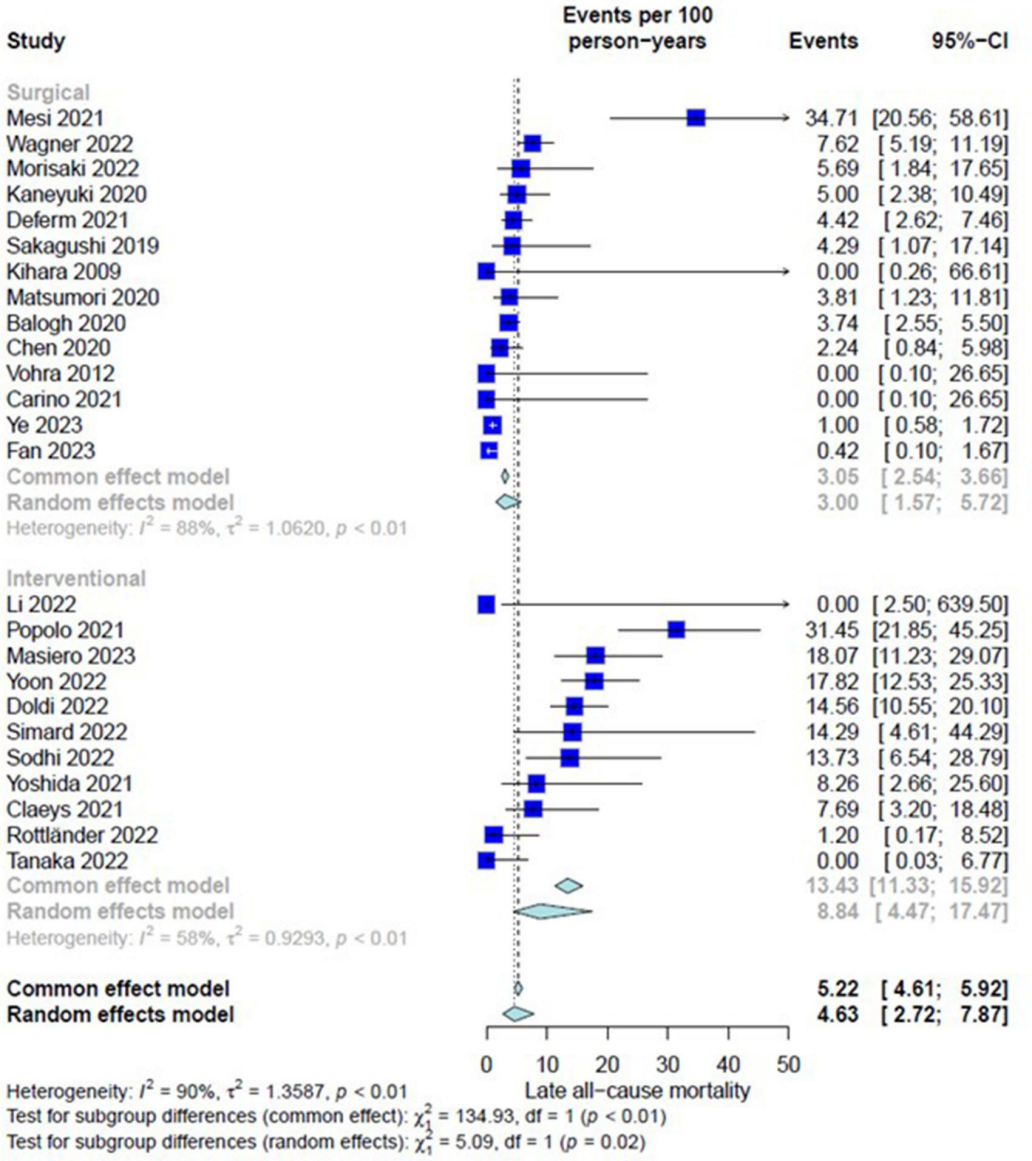
Forest Plot, Subgroup Differences Between Surgery Group and Transcatheter Edge To Edge Repair Group for Incidence Rate of Late (Follow-Up) All-Cause Mortality.

Twenty-five studies (1617 patients) were assessed. Overall IR of the entire group was 4.63 event per 100 PY (CI = 2.72-7.87). There was significant heterogeneity among included studies (*I*^2^ = 58%, *P *< .01). Subgroup analysis showed significantly higher IR of late all-cause mortality in the TEER group (3.00 [1.57-5.72] in surgery vs 8.84 [4.47-17.47] in TEER, interaction-*P* = .02) (**Figure 2**). On meta-regression, significant associations were found between increased IR of late all-cause mortality and both advanced age (β = 0.0750 [0.0338], *P *= .0263) and preprocedural hypertension (β = 0.0365 [0.0087], *P *< .001) across the entire cohort (**Table 3**). Within the surgical group, preprocedural hypertension (β = 0.0343 [0.0090], *P *< .001), MVR (β = 0.0508 [0.0131], *P *< .001), and associated AV surgery were significantly linked to an increased IR of late all-cause mortality. In the TEER group, hypertension was the sole variable associated with an increased IR of late all-cause mortality (β = .2034 [0.1057], *P *= .0542) (**Table 3**).

##### Late HF hospitalization/readmission

**Figure 3.**
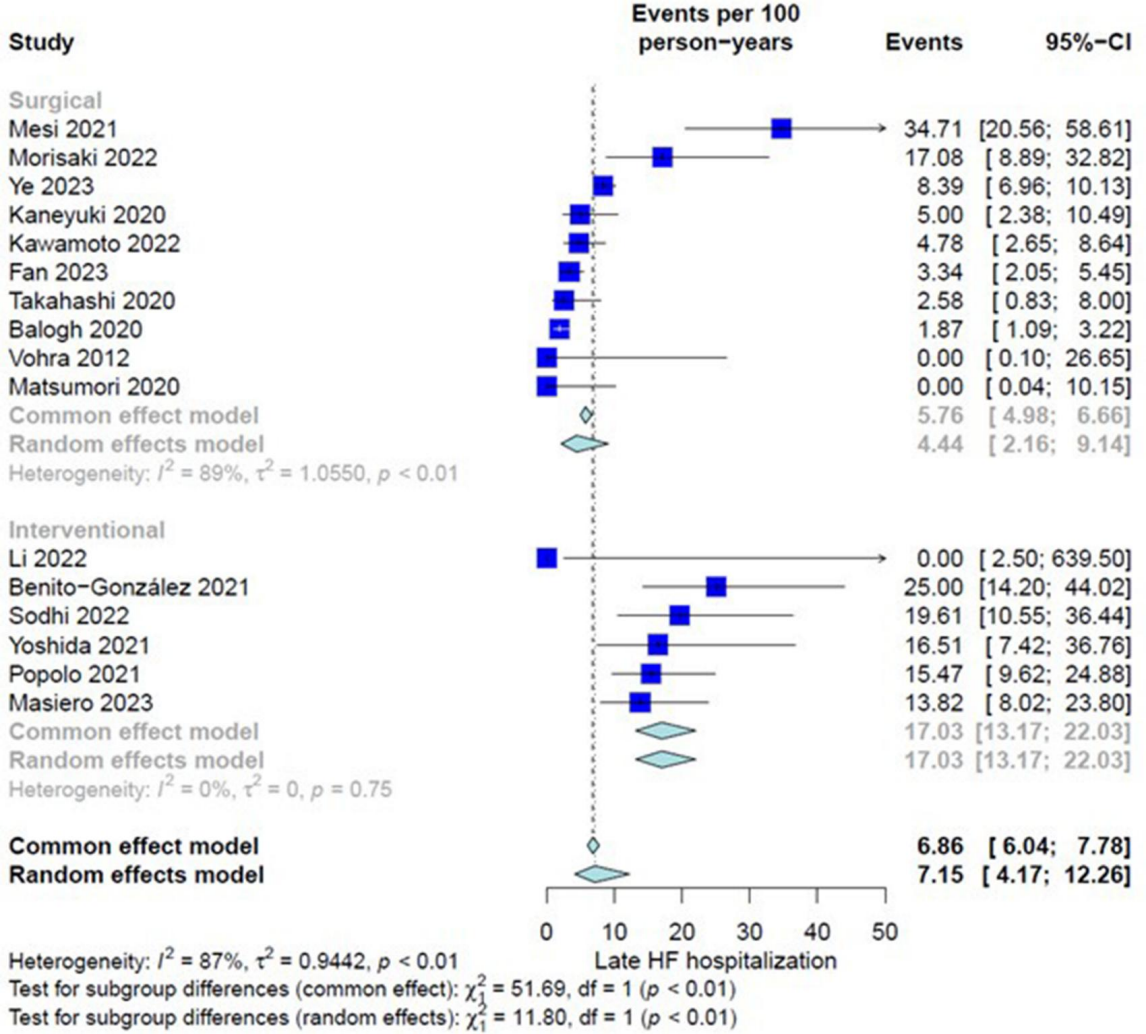
Forest Plot, Subgroup Differences Between Surgery Group and Transcatheter Edge To Edge Repair (TEER) Group for Incidence Rate of Late (Follow-Up) Heart Failure (HF) Hospitalization/Re-Admission.

Sixteen studies (968 patients) were assessed. Overall IR in the entire group was 7.15 events per 100 PY (CI = 4.17-12.26). There was significant heterogeneity among included studies (*I*^2^ = 87%, *P *< .01). Subgroup analysis showed a significantly higher IR of late HF readmission in the TEER group (4.44 [2.16-9.14] in surgery vs 17.03 [13.17-22.03] in TEER, interaction-*P *< .01) (**Figure 3**).

##### Late postprocedural NYHA III/IV

**Figure 4.**
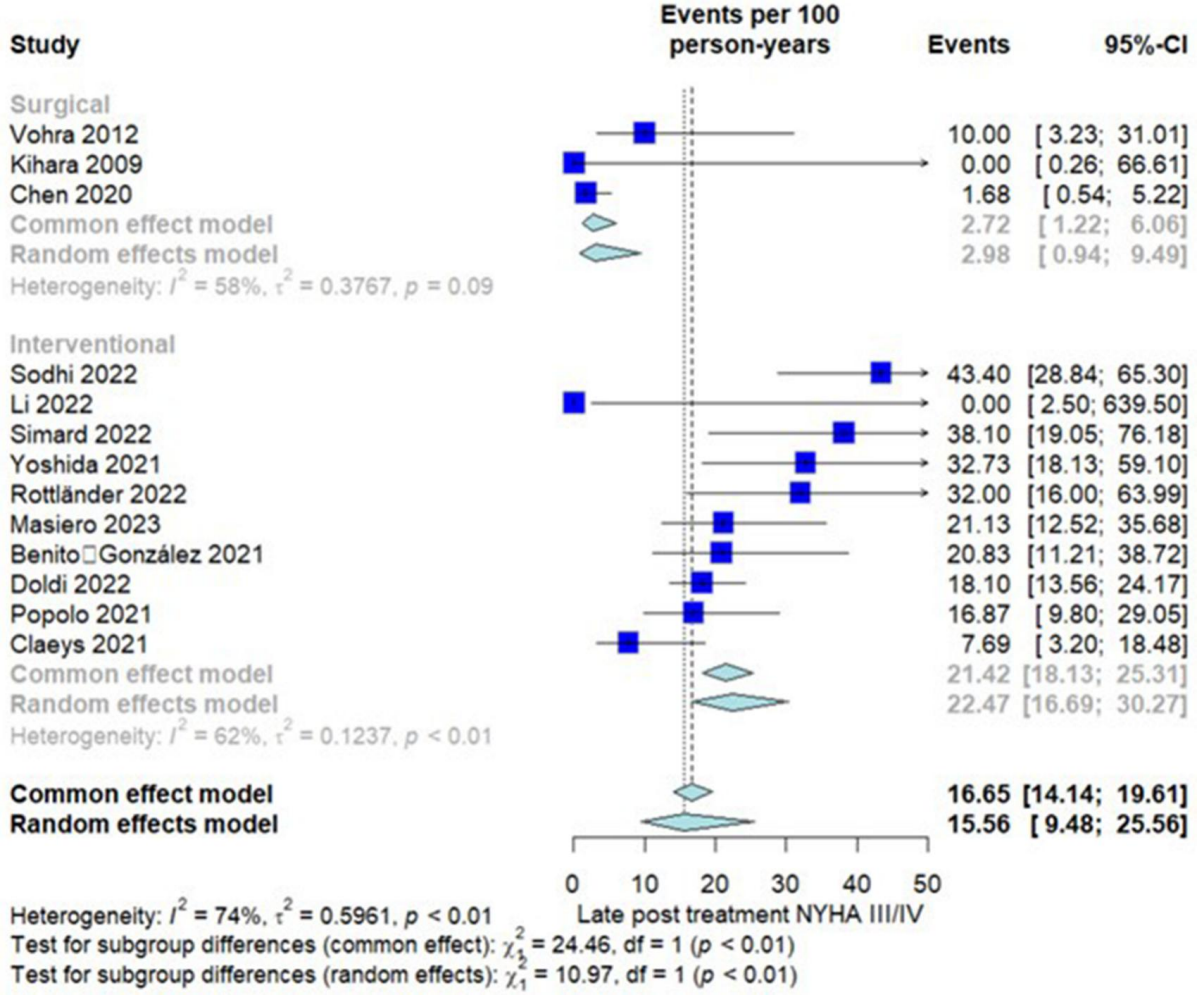
Forest Plot, Subgroup Differences Between Surgery Group and Transcatheter Edge To Edge Repair Group for Incidence Rate of Late (Follow-Up) Postprocedural New York Heart Association Classification (NYHA) III/IV.

Thirteen studies (587 patients) were assessed. Overall IR in the entire group was 15.56 event per 100 PY (CI = 9.48-25.56). There was significant heterogeneity among included studies (*I*^2^ = 96%, *P *< .01). Subgroup analysis showed significantly higher IR of late postprocedural NYHA III/IV in the TEER group (2.98 [0.94-9.49] in surgery vs 22.47 [16.69-30.27] in TEER, interaction-*P *= .01) (**Figure 4**).

##### Early AKI

Thirteen studies (650 patients) were assessed. Overall pooled events in the entire cohort were 4.09 events per 100 PY (CI = 2.38-6.94). There was mild heterogeneity among included studies (*I*^2^ = 17%, *P *= .27). Subgroup analysis showed no significant difference between groups (interaction-*P *= .0504) (**Figure S3**).

##### Early all-cause mortality

Thirty studies (1860 patients) were assessed. Overall pooled events in the entire cohort were 2.37 events per 100 PY (CI = 1.68-3.34). There was no heterogeneity among included studies (*I*^2^ = 0%, *P *= .84). Subgroup analysis did not show a significant difference between groups (interaction-*P *= .0515) (**Figure S4).** 

##### Early cardiac-specific mortality

Twenty-six studies (1418 patients) were assessed. Overall pooled events in the entire cohort were 1.92 event per 100 PY (CI = 1.22-3.02). There was no heterogeneity among included studies (*I*^2^ = 0%, *P *= .95). Subgroup analysis did not show significant difference between groups (interaction-*P *= .33) (**Figure S5**).

##### Early stroke

Sixteen studies (1031 patients) were assessed. Overall pooled events in the entire cohort were 2.62 events per 100 PY (CI = 1.72-3.98). There was no heterogeneity among included studies (*I*^2^ = 0%, *P *= .72). Subgroup analysis showed no significant difference between groups (interaction-*P *= .68) (**Figure S6**).

##### Early HF hospitalization

Eighteen studies (940 patients) were assessed. Overall pooled events in the entire cohort were 3.33 events per 100 PY (CI = 1.83-5.99). There was significant heterogeneity among included studies (*I*^2^ = 44%, *P *= .02). Subgroup analysis did not show significant difference between groups (interaction-*P *= .96) (**Figure S7**).

##### Early reoperation/reintervention

Twenty studies (969 patients) were assessed. Overall pooled events in the entire cohort were 2.96 events per 100 PY (CI = 1.85-4.69). There was no significant heterogeneity among included studies (*I*^2^ = 2%, *P *= .44). Subgroup analysis did not show significant difference between groups (interaction-*P *= .22) (**Figure S8**).

#### Postprocedural atrial and ventricular reverse remodelling

##### Mean left atrial diameter (mm)

Ten studies (555 patients) were assessed. There was significant heterogeneity among included studies (*I*^2^ = 87%, *P *< .01). Subgroup analysis did not show significant difference between groups (50.45 [CI = 48.11-52.79] in surgery vs 56.15 [CI = 44.64-67.66] in TEER, interaction-*P *= .34) (**Figure S9**).

##### Mean LVESD (mm)

Ten studies (705 patients) were assessed. There was significant heterogeneity among included studies (*I*^2^ = 89%, *P *< .01). Subgroup analysis did not show significant difference between groups (33.94 [32.36-35.52] in surgery vs 30.42 [24.01-36.83] in TEER, interaction-*P *= .30) (**Figure S10**).

##### Mean LVEDD (mm)

Thirteen studies (776 patients) were assessed for this outcome. There was significant heterogeneity among the included studies (*I*^2^ = 94%, *P* < .01) (**Figure S11**). Subgroup analysis did not show any significant difference between both groups (49.18 [47.48-50.88] in surgery vs 57.78 [40.43-75.12] in TEER, interaction-*P* = .33) (**Figure S11**).

#### Postprocedural mean EF

Fifteen studies (1014 patients) were assessed. Mean EF in the entire cohort was 57.27 (55.38-59.15) with significant heterogeneity among included studies (*I*^2^ = 95%, *P *< .01). Subgroup analysis did not show significant difference between groups (57.03 [54.80-59.26] in surgery vs 57.60 [54.07-61.13] in TEER, interaction-*P *= .79) (**Figure S12**).

#### Late reoperation

Seventeen studies (690 patients) were assessed. Overall IR in the entire group was 1.62 events per 100 PY (CI = 1.03-2.55). There was no significant heterogeneity among included studies (*I*^2^ = 0%, *P *= .52). There was no significant difference between groups in the subgroup analysis (interaction-*P *= .17) (**Figure S13**).

#### Late cardiac-specific mortality

Sixteen studies (859 patients) were assessed. Overall IR in the entire group was 1.36 events per 100 PY (CI = 0.54-3.45). There was significant heterogeneity among included studies (*I*^2^ = 80%, *P *= .01). There was no significant difference between groups in the subgroup analysis (interaction-*P *= .23) (**Figure S14**).

#### Late stroke

Thirteen studies (884 patients) were assessed. Overall IR in the entire group was 0.51 events per 100 PY (CI = 0.32-0.83). There was significant heterogeneity among included studies (*I*^2^ = 64%, *P *< .01). There was no significant difference between groups in the subgroup analysis (interaction-*P *= .35) (**Figure S15**).

## DISCUSSION

To the best of our knowledge, this is the only meta-analysis comparing early and late outcomes of surgical and transcatheter MV interventions for AFMR. Our main findings were that, compared to isolated mitral TEER, surgical intervention (including mitral annuloplasty, concomitant AF ablation, and/or TV repair) was associated with decreased IR of:

1. Late severe MR (2.53 vs 6.66 events per 100 PY, *P*-interaction = .03)
2. Late all-cause mortality (3.00 vs 8.84 events per 100 PY, *P*-interaction = .024)
3. Late HF hospitalization/readmission (4.44 vs 17.03 events per 100 PY, *P*-interaction < .01)
4. Late NYHA III/IV (2.98 vs 22.47 events per 100 PY, *P*-interaction < .01)
5. Both groups were comparable in IR of early all-cause mortality, early cardiac-specific mortality, late cardiac-specific mortality, postprocedural morbidities (AKI and early stroke), and postprocedural atrial and ventricular reverse remodelling.

We attribute the better outcome of surgical intervention over TEER to concomitant interventions such as *mitral annuloplasty*, AF ablation, and TR repair that address other aspects of AFMR pathophysiology including atrial fibrillation and TR.

### Early and late severe mitral regurgitation

Our study demonstrated that IR of early and late recurrent MR was significantly higher in TEER compared to surgery. An explanation is that surgery directly repairs annular dilation, the primary cause of AFMR, while TEER only approximates leaflets, and its effect on the annulus is not as direct as surgery.

Several studies have reported mitral annular diameter decrease following TEER, particularly in patients with FMR,^22–27^ supporting the treatment rationale. Patzelt *et al.* reported a reduction in mitral annular diameters in VFMR at patient follow-up^28^; however, we could not analyse this reduction due to data unavailability. Therapeutic left ventricular remodelling after TEER can also be assessed by a reduction in LVEDD.^29^ Our analysis showed both interventions resulted in comparable reductions in left atrial and left ventricular diameters, with slight benefit associated with the surgical group despite their relatively larger preprocedural left atrial and left ventricular diameters and volumes. These findings further suggest that superior outcomes associated with surgery may stem from its direct repair of the mitral annulus, while TEER relies on delayed cardiac remodelling.

To our knowledge, no comparative studies or randomized controlled trials (RCTs) have directly compared surgery to TEER for AFMR, as opposed to VFMR. The EVEREST II trial^30^^,^^31^ is the only trial comparing TEER to surgical interventions, but for different aetiologies of MR. The trial reported superior safety and similar improvements in clinical outcomes for TEER, despite less MR reduction than surgery. In a subgroup of patients with unspecified FMR, freedom from death, MV surgery, and MR grade ≥3+ was 54% in TEER vs 50% in surgery (*P *= .02). At 5 years, the incidence of 3+/4+ MR and surgery for MV dysfunction increased in the TEER group, particularly during the first year.^32^ Despite different aetiologies for MR between trials, our results align with those of the EVEREST II trial regarding the incidence of late recurrent MR and safety of the TEER procedure. The EVEREST II trial and our results suggest that, regardless of baseline comorbidities, surgery offers better outcomes. It is well documented that surgical outcomes are generally better for AFMR compared to VFMR with regard to operative and all-cause mortality, survival, and MR recurrence,^33^^,^^34^ whereas superior results of TEER vs surgery in the VFMR group have been reported.^35^ However, our meta-analysis suggests that surgical outcomes are better for AFMR than for TEER group. We believe that differences in TEER outcomes between VFMR and AFMR stem from anatomical and pathophysiological differences. Mitral leaflets in VFMR often have tethering and an anatomic target for TEER, while AFMR generally has MR across the entire coaptation plane without a focused target. Additionally, follow-up period for the TEER group in our meta-analysis was only 12 months, leaving the possibility of outcome improvement with longer follow-up after TEER. Another concern is the different generations of TEER devices used across various studies and enrolment periods, as there have been technological advancements linked to improved outcomes and durability, as indicated by the EXPAND study.^36^ The novel PASCAL (Precision Transcatheter Valve Repair System, Edwards Lifesciences, Irvine, CA) system has not yet been used for AFMR,^37^ and there have been no direct comparative studies between Mitra-Clip (Abbott, Santa Clara, CA) and PASCAL until the ongoing Clinical Long-term Assessment of the Safety and Performance of the PASCAL System (CLASP) IIF RCT in FMR patients (NCT03706833) that might introduce more definitive data.

### Associated AF ablation procedures

In our study, approximately 89% of the surgical group and 86% of the TEER group had preprocedural AF. Most patients in the surgical group underwent concomitant AF ablation with or without left atrial appendage exclusion (863/1162, 74.3%), without any in the TEER group where the majority received medical treatment for AF (**Tables S7 and S8**). It is well established that AF is a significant predictor of worse early and late survival after surgical repair of MR,^38^^,^^39^ regardless of aetiology,^40^ and is associated with subsequent HF deterioration,^41^ even after TEER.^42-44^

A meta-analysis involving 7678 patients revealed that persistent AF after TEER was associated with increased risks of 1-year all-cause mortality, HF hospitalization, and bleeding.^42^ Although medical management of AF was associated with an equivalent reduction in MR severity compared to transcatheter ablation,^45^ many studies revealed superior long-term outcomes with transcatheter ablation.^2^^,^^46–50^ The CAMERA-MRI RCT reports an improvement in left ventricular ejection fraction (LVEF) with transcatheter AF ablation compared with medical AF management (16.4% vs 8.6%, *P *= .001) in patients with AF and systolic dysfunction.^48^ In patients with FMR, transcatheter ablation demonstrated similar results, along with a significant reduction in MR severity.^47^ Moreover, combining surgery with transcatheter ablation demonstrated greater reduction in MR severity and a decrease in late HF readmissions as compared with transcatheter ablation alone.^51^

Based on prior studies, lack of proper AF control after TEER may explain the superior outcomes of surgery, given the feasibility, safety, and good outcomes of transcatheter ablation after TEER as reported by Rottner *et al*.^52^ This underscores the importance of rhythm control in patients with AF undergoing mitral valve interventions, whether surgical or percutaneous.

### Associated tricuspid repair procedures

Our analysis revealed that 71.58% of the surgical group and 56.18% of the TEER group had concomitant TR >2+. AFMR is frequently accompanied by atrial functional TR (AFTR).^40^^,^^53–57^ Many studies report that moderate to severe TR adversely affect survival, independent of EF or pulmonary artery systolic pressure,^58–61^ particularly in patients with impaired LVEF, but not for those with end-stage HF.^62^ Despite poor prognosis and the class IIa indication by the American College of Cardiology/American Heart Association valve disease guidelines for patients with symptomatic severe AFTR,^11^ open-heart repair was seldom performed for isolated AFTR due to concerns regarding mortality.^63^ There is currently no consensus on optimal timing for intervention in AFTR, given the paucity of data regarding TR severity, annular size, or right ventricular (RV) function.^64^ Another important note is, surgical referral is often delayed until the condition progresses to severe right ventricular dysfunction adding to higher procedural risks.^64^ However, many studies recommend TR repair, for its greater benefits with intervention before the onset of RV, hepatic, and/or renal dysfunction.^64^

In addition, recent evidence suggests that rhythm control for AF via transcatheter ablation and/or electrical cardioversion can significantly reduce TR severity.^65^ The role of percutaneous interventions for AFTR is still evolving, data from the international multicentre TriValve registry demonstrate the safety and feasibility of percutaneous interventions.^57^ Procedural success was achieved in 62% of cases, with all-cause mortality of 3.7% and major adverse cardiac and cerebrovascular events in 26% of patients, and 58% of patients improved to NYHA class I or II at 30 days.^57^

In this meta-analysis, we did not find explanations of the absence of TR intervention nor AF ablative procedure in the TEER group despite the established efficacy and safety of such procedures; This could have accounted for the superior outcomes in the surgical group (746/1165, 64.0% in the surgery group underwent concomitant TR repair). This highlights the need for a more comprehensive approach in managing concomitant TR in AFMR patients.

### Limitations

First, there is variability in the definitions,^6^ inclusion criteria,^6^ and echocardiographic characteristics^5^ across the included studies in both groups. Also, missing data and inconsistencies in reporting among the included studies hindered our ability to conduct subgroup analyses. Moreover, there were limitations related to the meta-analytical approach itself, as there were no direct comparative studies between both approaches. Another limitation is the heterogeneity of the baseline preoperative characteristics between both groups. Most patients in the TEER group *were older*, had more associated comorbidities and higher STS-PROM and EuroSCORE II values, which may have introduced selection bias and affect outcomes.

Advancement of technology, especially TEER devices, may have influenced outcomes. Different generations of Mitra-Clip were used in the included TEER studies, which could have affected the results.

Also, the follow-up duration for the TEER group was significantly shorter. This constraint prevents us from assessing longer-term outcomes. Lastly, heterogeneity among the surgical techniques employed in the surgical group may have contributed to the observed differences in outcomes. Because of these limitations, it is difficult to arrive at definitive conclusions from this limited data, and the findings from this study are perhaps best regarded as hypothesis-generating.

## CONCLUSION AND RECOMMENDATION

Based on the results of this meta-analysis, both surgical intervention and TEER for AFMR confer comparable results regarding short-term outcomes (early HF hospitalization, early mortality, and early intervention). *TEER patients were older and had higher comorbidity indices*. However, surgery appears to be superior to TEER in terms of immediate reduction in MR severity and beneficial long-term outcomes, including lower rates of late recurrent severe MR, HF hospitalization, all-cause mortality, and NYHA Class III and IV symptoms. This might be attributed to correction of *mitral annular dilatation*, more complete AF ablation, intervention to TR when necessary, and *more adverse comorbidities of the TEER group*. However, given the proven feasibility, safety, and effectiveness of transcatheter approaches, whether combining transcatheter TR repair and AF ablation with MR TEER could potentially enhance the outcomes of TEER vs surgery is not known and should be further tested and explored in clinical practice.

## Supplementary Material

ivaf269_Supplementary_Data

## Data Availability

All relevant data are within the manuscript and its Supporting Information files.

## References

[1] Asgar AW , Mack MJ, Stone GW. Secondary mitral regurgitation in heart failure: pathophysiology, prognosis, and therapeutic considerations. J Am Coll Cardiol. 2015;65:1231-1248. 25814231 10.1016/j.jacc.2015.02.009

[2] Gertz ZM , Raina A, Saghy L, et al Evidence of atrial functional mitral regurgitation due to atrial fibrillation: reversal with arrhythmia control. J Am Coll Cardiol. 2011;58:1474-1481. 21939832 10.1016/j.jacc.2011.06.032

[3] Abe Y , Takahashi Y, Shibata T. Functional mitral regurgitation, updated: ventricular or atrial? J Echocardiogr. 2020;18:1-8. 31728977 10.1007/s12574-019-00453-w

[4] Reid A , Zekry SB, Naoum C, et al Geometric differences of the mitral valve apparatus in atrial and ventricular functional mitral regurgitation. J Cardiovasc Comput Tomogr. 2022;16:431-441. 35361564 10.1016/j.jcct.2022.02.008

[5] Farhan S , Silbiger JJ, Halperin JL, et al Pathophysiology, echocardiographic diagnosis, and treatment of atrial functional mitral regurgitation: JACC state-of-the-art review. J Am Coll Cardiol. 2022;80:2314-2330. 36480974 10.1016/j.jacc.2022.09.046

[6] Amabile A , Fereydooni S, Mori M, et al Variable definitions and treatment approaches for atrial functional mitral regurgitation: a scoping review of the literature. J Card Surg. 2022;37:1182-1191. 35179258 10.1111/jocs.16312

[7] Schnabel RB , Yin X, Gona P, et al 50 Year trends in atrial fibrillation prevalence, incidence, risk factors, and mortality in the Framingham Heart Study: a cohort study. Lancet. 2015;386:154-162. 25960110 10.1016/S0140-6736(14)61774-8 PMC4553037

[8] Chen J , Wang Y, Lv M, et al Mitral valve repair and surgical ablation for atrial functional mitral regurgitation. Ann Transl Med. 2020;8:1420. 33313165 10.21037/atm-20-2958 PMC7723636

[9] Deferm S , Bertrand PB, Verbrugge FH, et al Atrial functional mitral regurgitation: JACC review topic of the week. J Am Coll Cardiol. 2019;73:2465-2476. 31097168 10.1016/j.jacc.2019.02.061

[10] Stone GW , Lindenfeld J, Abraham WT, et al; COAPT Investigators. Transcatheter mitral-valve repair in patients with heart failure. N Engl J Med. 2018;379:2307-2318. 30280640 10.1056/NEJMoa1806640

[11] Members WC , Otto CM, Nishimura RA, et al 2020 ACC/AHA guideline for the management of patients with valvular heart disease: a report of the American College of Cardiology/American Heart Association Joint Committee on Clinical Practice Guidelines. J Am Coll Cardiol. 2021;77:e25-e197. 33342586 10.1016/j.jacc.2020.11.018

[12] Vahanian A , Beyersdorf F, Praz F, et al; ESC/EACTS Scientific Document Group. 2021 ESC/EACTS guidelines for the management of valvular heart disease: developed by the task force for the management of valvular heart disease of the European Society of Cardiology (ESC) and the European Association for Cardio-Thoracic Surgery (EACTS). Eur Heart J. 2022;43:561-632. 35188539 10.1093/eurheartj/ehac051

[13] Izumi C , Eishi K, Ashihara K, et al; Japanese Circulation Society Joint Working Group. JCS/JSCS/JATS/JSVS 2020 guidelines on the management of valvular heart disease. Circulation Journal. 2020;84:2037-2119. 32921646 10.1253/circj.CJ-20-0135

[14] Page MJ , McKenzie JE, Bossuyt PM, et al The PRISMA 2020 statement: an updated guideline for reporting systematic reviews. BMJ. 2021;372. 10.1136/bmj.n71 PMC8005924 33782057

[15] Wells GA , Shea B, O’Connell D, et al *The Newcastle-Ottawa Scale (NOS) for Assessing the Quality of Nonrandomised Studies in Meta-analyses*. Oxford; 2000.

[16] Viechtbauer W. Conducting meta-analyses in R with the metafor package. J Stat Soft. 2010;36:1-48.

[17] Balduzzi S , Rücker G, Schwarzer G. How to perform a meta-analysis with R: a practical tutorial. Evid Based Mental Health. 2019;22:153-160. 10.1136/ebmental-2019-300117 PMC10231495 31563865

[18] Guyot P , Ades AE, Ouwens MJ, Welton NJ. Enhanced secondary analysis of survival data: reconstructing the data from published Kaplan-Meier survival curves. BMC Med Res Methodol. 2012;12:9. 10.1186/1471-2288-12-9 22297116 PMC3313891

[19] Gaudino M , Rahouma M, Di Mauro M, et al Early versus delayed stroke after cardiac surgery: a systematic review and meta-analysis. J Am Heart Assoc. 2019;8:e012447. 10.1161/jaha.119.012447 31215306 PMC6662344

[20] Gaudino M , Girardi LN, Rahouma M, et al Editor’s choice—aortic Re-operation after replacement of the proximal aorta: a systematic review and meta-analysis. Eur J Vasc Endovasc Surg. 2018;56:515-523. 10.1016/j.ejvs.2018.06.038 30037741

[21] Kamal M , Baudo M, Shmushkevich S, et al COVID-19 infection and its consequences among surgical oncology patients: a systematic analysis, meta-analysis and meta-regression. J Surg Oncol. 2022;125:813-823. 10.1002/jso.26787 35014703 PMC9015254

[22] Paukovitsch M , Felbel D, Jandek M, et al Transcatheter edge-to-edge-repair of functional mitral regurgitation induces significant remodeling of mitral annular geometry. Front Cardiovasc Med. 2023;10:1143702. 37424917 10.3389/fcvm.2023.1143702 PMC10326617

[23] Schueler R , Momcilovic D, Weber M, et al Acute changes of mitral valve geometry during interventional edge-to-edge repair with the MitraClip system are associated with midterm outcomes in patients with functional valve disease: preliminary results from a prospective single-center study. Circ Cardiovasc Interv. 2014;7:390-399. 24895448 10.1161/CIRCINTERVENTIONS.113.001098

[24] Kim J , Palumbo MC, Khalique OK, et al Transcatheter MitraClip repair alters mitral annular geometry–device induced annular remodeling on three-dimensional echocardiography predicts therapeutic response. Cardiovasc Ultrasound. 2019;17:31-11. 31878931 10.1186/s12947-019-0181-z PMC6933704

[25] Schueler R , Kaplan S, Melzer C, et al Impact of interventional edge-to-edge repair on mitral valve geometry. Int J Cardiol. 2017;230:468-475. 28041699 10.1016/j.ijcard.2016.12.081

[26] Schmidt FP , von Bardeleben RS, Nikolai P, et al Immediate effect of the MitraClip procedure on mitral ring geometry in primary and secondary mitral regurgitation. Eur Heart J Cardiovasc Imaging. 2013;14:851-857. 23288891 10.1093/ehjci/jes293

[27] Noack T , Kiefer P, Mallon L, et al Changes in dynamic mitral valve geometry during percutaneous edge-edge mitral valve repair with the MitraClip system. J Echocardiogr. 2019;17:84-94. 10.1007/s12574-018-0398-0 30291509

[28] Patzelt J , Zhang Y, Magunia H, et al Improved mitral valve coaptation and reduced mitral valve annular size after percutaneous mitral valve repair (PMVR) using the MitraClip system. Eur Heart J Cardiovasc Imaging. 2018;19:785-791. 10.1093/ehjci/jex173 28977372

[29] Kim J , Alakbarli J, Palumbo MC, et al Left ventricular geometry predicts optimal response to percutaneous mitral repair via MitraClip: integrated assessment by two‐ and three‐dimensional echocardiography. Catheter Cardiovasc Interv. 2019;93:1152-1160. 30790417 10.1002/ccd.28147 PMC6537596

[30] Mauri L , Garg P, Massaro JM, et al The EVEREST II trial: design and rationale for a randomized study of the evalve mitraclip system compared with mitral valve surgery for mitral regurgitation. Am Heart J. 2010;160:23-29. 10.1016/j.ahj.2010.04.009 20598968

[31] Mauri L , Foster E, Glower DD, et al; EVEREST II Investigators. 4-year results of a randomized controlled trial of percutaneous repair versus surgery for mitral regurgitation. J Am Coll Cardiol. 2013;62:317-328. 10.1016/j.jacc.2013.04.030 23665364

[32] Feldman T , Kar S, Elmariah S, et al Randomized comparison of percutaneous repair and surgery for mitral regurgitation: 5-Year results of EVEREST II. J Am Coll Cardiol. 2015;66:2844-2854. 10.1016/j.jacc.2015.10.018 26718672

[33] Hirji SA , Cote CL, Javadikasgari H, Malarczyk A, McGurk S, Kaneko T. Atrial functional versus ventricular functional mitral regurgitation: prognostic implications. J Thorac Cardiovasc Surg. 2022;164:1808-1815.e4. 10.1016/j.jtcvs.2020.12.098 33526277

[34] Deferm S , Bertrand PB, Verhaert D, et al Outcome and durability of mitral valve annuloplasty in atrial secondary mitral regurgitation. Heart. 2021;107:1503-1509. 10.1136/heartjnl-2021-319045 34415852

[35] Felbel D , Paukovitsch M, Förg R, et al Comparison of transcatheter edge-to-edge and surgical repair in patients with functional mitral regurgitation using a meta-analytic approach. Front Cardiovasc Med. 2022;9:1063070. 10.3389/fcvm.2022.1063070 36762304 PMC9905105

[36] Mahoney PD , Price M, Rinaldi MJ, et al The evolution of transcatheter edge to edge repair with mitraclip and its outcomes in secondary mitral regurgitation. J Am Coll Cardiol. 2022;79:578-578.

[37] Srinivasan A , Brown J, Ahmed H, Daniel M. PASCAL repair system for patients with mitral regurgitation: a systematic review. Int J Cardiol. 2023;376:108-114. 10.1016/j.ijcard.2023.01.026 36681242

[38] Alexiou C , Doukas G, Oc M, et al The effect of preoperative atrial fibrillation on survival following mitral valve repair for degenerative mitral regurgitation. Eur J Cardiothorac Surg. 2007;31:586-591. 17280837 10.1016/j.ejcts.2006.12.039

[39] Grigioni F , Avierinos J-F, Ling LH, et al Atrial fibrillation complicating the course of degenerative mitral regurgitation: determinants and long-term outcome. J Am Coll Cardiol. 2002;40:84-92. 12103260 10.1016/s0735-1097(02)01922-8

[40] Abe Y , Akamatsu K, Ito K, et al Prevalence and prognostic significance of functional mitral and tricuspid regurgitation despite preserved left ventricular ejection fraction in atrial fibrillation patients. Circ J. 2018;82:1451-1458. 29553091 10.1253/circj.CJ-17-1334

[41] Chung MK , Shemanski L, Sherman DG, et al; AFFIRM Investigators. Functional status in rate-versus rhythm-control strategies for atrial fibrillation: results of the atrial fibrillation Follow-Up investigation of rhythm management (AFFIRM) functional status substudy. J Am Coll Cardiol. 2005;46:1891-1899. 16286177 10.1016/j.jacc.2005.07.040

[42] Shah S , Raj V, Abdelghany M, et al Impact of atrial fibrillation on the outcomes of transcatheter mitral valve repair using MitraClip: a systematic review and meta-analysis. Heart Fail Rev. 2021;26:531-543. 33169339 10.1007/s10741-020-10051-z

[43] Keßler M , Pott A, Mammadova E, et al Atrial fibrillation predicts long-term outcome after transcatheter edge-to-edge mitral valve repair by MitraClip implantation. Biomolecules. 2018;8:152. 30463247 10.3390/biom8040152 PMC6316291

[44] Velu JF , Kortlandt FA, Hendriks T, et al Comparison of outcome after percutaneous mitral valve repair with the MitraClip in patients with versus without atrial fibrillation. Am J Cardiol. 2017;120:2035-2040. 29033048 10.1016/j.amjcard.2017.08.022

[45] Ito K , Abe Y, Watanabe H, et al Prognostic significance of residual functional mitral regurgitation in hospitalized heart failure patients with chronic atrial fibrillation and preserved ejection fraction after medical therapies. J Echocardiogr. 2019;17:197-205. 30569445 10.1007/s12574-018-0412-6

[46] Wu J-T , Zaman JA, Yakupoglu HY, et al Catheter ablation of atrial fibrillation in patients with functional mitral regurgitation and left ventricular systolic dysfunction. Front Cardiovasc Med. 2020;7:596491. 33381527 10.3389/fcvm.2020.596491 PMC7767831

[47] Wu J-T , Zhao D-Q, Zhang F-T, et al Effect of catheter ablation on clinical outcomes in patients with atrial fibrillation and significant functional mitral regurgitation. BMC Cardiovasc Disord. 2021;21:587-510. 34876011 10.1186/s12872-021-02397-5 PMC8650368

[48] Sugumar H , Prabhu S, Costello B, et al Catheter ablation versus medication in atrial fibrillation and systolic dysfunction: late outcomes of CAMERA-MRI study. JACC Clin Electrophysiol. 2020;6:1721-1731. 33334453 10.1016/j.jacep.2020.08.019

[49] Marrouche NF , Brachmann J, Andresen D, et al; CASTLE-AF Investigators. Catheter ablation for atrial fibrillation with heart failure. N Engl J Med. 2018;378:417-427. 29385358 10.1056/NEJMoa1707855

[50] Kuck K-H , Merkely B, Zahn R, et al Catheter ablation versus best medical therapy in patients with persistent atrial fibrillation and congestive heart failure: the randomized AMICA trial. Circ Arrhythm Electrophysiol. 2019;12:e007731. 31760819 10.1161/CIRCEP.119.007731

[51] Fan X , Tang Y, Ma Y, et al Mitral valve repair and concomitant maze procedure versus catheter ablation in the treatment of atrial functional mitral regurgitation. BMC Cardiovasc Disord. 2022;22:543. 10.1186/s12872-022-02972-4 36510122 PMC9743701

[52] Rottner L , Lemes C, Dotz I, et al The clip and the tip: lessons learned from ablation of atrial fibrillation in patients postpercutaneous mitral valve repair. J Cardiovasc Electrophysiol. 2019;30:1207-1214. 31045293 10.1111/jce.13964

[53] Guta AC , Badano LP, Tomaselli M, et al The pathophysiological link between right atrial remodeling and functional tricuspid regurgitation in patients with atrial fibrillation: a three-dimensional echocardiography study. J Am Soc Echocardiogr. 2021;34:585-594. e1. 33440232 10.1016/j.echo.2021.01.004

[54] Mesi O , Gad MM, Crane AD, et al Severe atrial functional mitral regurgitation: clinical and echocardiographic characteristics, management and outcomes. JACC Cardiovasc Imaging. 2021;14:797-808. 33832663 10.1016/j.jcmg.2021.02.008

[55] Ortiz-Leon XA , Posada-Martinez EL, Trejo-Paredes MC, et al Tricuspid and mitral remodelling in atrial fibrillation: a three-dimensional echocardiographic study. Eur Heart J Cardiovasc Imaging. 2022;23:944-955. 35243501 10.1093/ehjci/jeac045

[56] Taramasso M , Vanermen H, Maisano F, Guidotti A, La Canna G, Alfieri O. The growing clinical importance of secondary tricuspid regurgitation. J Am Coll Cardiol. 2012;59:703-710. 22340261 10.1016/j.jacc.2011.09.069

[57] Taramasso M , Hahn RT, Alessandrini H, et al The international multicenter TriValve registry: which patients are undergoing transcatheter tricuspid repair? JACC Cardiovasc Interv. 2017;10:1982-1990. 28982563 10.1016/j.jcin.2017.08.011

[58] Nath J , Foster E, Heidenreich PA. Impact of tricuspid regurgitation on long-term survival. J Am Coll Cardiol. 2004;43:405-409. 15013122 10.1016/j.jacc.2003.09.036

[59] Delling FN , Hassan ZK, Piatkowski G, et al Tricuspid regurgitation and mortality in patients with transvenous permanent pacemaker leads. Am J Cardiol. 2016;117:988-992. 26833208 10.1016/j.amjcard.2015.12.038 PMC4775321

[60] Messika-Zeitoun D , Thomson H, Bellamy M, et al Medical and surgical outcome of tricuspid regurgitation caused by flail leaflets. J Thorac Cardiovasc Surg. 2004;128:296-302. 15282468 10.1016/j.jtcvs.2004.01.035

[61] Takahashi Y , Izumi C, Miyake M, et al Actual management and prognosis of severe isolated tricuspid regurgitation associated with atrial fibrillation without structural heart disease. Int J Cardiol. 2017;243:251-257. 28536002 10.1016/j.ijcard.2017.05.031

[62] Neuhold S , Huelsmann M, Pernicka E, et al Impact of tricuspid regurgitation on survival in patients with chronic heart failure: unexpected findings of a long-term observational study. Eur Heart J. 2013;34:844-852. 23335604 10.1093/eurheartj/ehs465

[63] Zack CJ , Fender EA, Chandrashekar P, et al National trends and outcomes in isolated tricuspid valve surgery. J Am Coll Cardiol. 2017;70:2953-2960. 29241483 10.1016/j.jacc.2017.10.039

[64] Fender EA , Zack CJ, Nishimura RA. Isolated tricuspid regurgitation: outcomes and therapeutic interventions. Heart. 2018;104:798-806. 29229649 10.1136/heartjnl-2017-311586 PMC5931246

[65] Soulat-Dufour L , Lang S, Addetia K, et al Restoring sinus rhythm reverses cardiac remodeling and reduces valvular regurgitation in patients with atrial fibrillation. J Am Coll Cardiol. 2022;79:951-961. 35272799 10.1016/j.jacc.2021.12.029

[66] Ye Q , Zhao Y, Bai C, et al Outcome of mitral repair combined with cox-maze procedure for atrial functional mitral regurgitation with heart failure with recovered ejection fraction. European J Cardiothorac Surg. 2023;64:ezad273. 37549058 10.1093/ejcts/ezad273

