# Supplementary material for: Comparison of Meta-Analytical Estimates Between Surgical Repair and Transcatheter Edge-to-Edge Repair for Atrial Functional Mitral Regurgitation
Source: Interdiscip Cardiovasc Thorac Surg. 2025 Dec 24;41(1):ivaf269. doi: 10.1093/icvts/ivaf269 (PMC12774469; doi:10.1093/icvts/ivaf269)
Supplement: ivaf269_Supplementary_Data [file ivaf269_supplementary_data.zip › Supplementary materials.docx]

**Supplementary materials**

**Supplementary Table S1**. Inclusion and exclusion criteria, and full search strategies for all databases.

**Ovid MEDLINE** (ALL – 1946 to present)

Searched on December 6, 2023

No language, article type, or publication date restrictions

| **Line #** | **Search** | **# of results** |
| --- | --- | --- |
| 1 | Atrial Fibrillation/ and (functional mitral or mitral regurgitation or mitral valve regurgitation).mp. | 790 |
| 2 | (atrial functional and (mitral regurgitation or mitral valve regurgitation)).mp. | 122 |
| 3 | (atrial fibrillation and (mitral regurgitation or mitral valve regurgitation)).mp. | 1802 |
| 4 | or/1-3 | 1835 |

**Ovid Embase** (1974 to present)

Searched on December 6, 2023

No language, article type, or publication date restrictions

| **Line #** | **Search** | **# of results** |
| --- | --- | --- |
| 1 | (atrial functional and (mitral regurgitation or mitral valve regurgitation)).mp. | 197 |
| 2 | (atrial fibrillation and (mitral regurgitation or mitral valve regurgitation)).tw | 3490 |
| 3 | 1 or 2 | 3578 |

**Cochrane Library** (Wiley)

Searched on December 6, 2023

No language, article type, or publication date restrictions

| ID | Search | Hits |
| --- | --- | --- |
| #1 | MeSH descriptor: [Atrial Fibrillation] explode all trees | 7504 |
| #2 | ("functional mitral" or "mitral regurgitation" or "mitral valve regurgitation"):ti,ab | 927 |
| #3 | #1 AND #2 | 35 |
| #4 | ("atrial functional" and ("mitral regurgitation" or "mitral valve regurgitation")):ti,ab | 3 |
| #5 | ("atrial fibrillation" and ("mitral regurgitation" or "mitral valve regurgitation")):ti,ab | 99 |
| #6 | #3 OR #4 OR #5 | 103 |

**Supplementary Table S2:** Inclusion and exclusion criteria

| Inclusion criteria |
| --- |
| Adults aged ≥18 years |
| Study population includes patients with AFMR^1^ |
| All AFMR etiologies: either anterior and/or posterior leaflet pseudo-prolapse or left atrial dilation with progressive annular dilatation |
| Surgical treatment and outcomes report: either open or minimally invasive techniques, annuloplasty alone or in combination with other procedures such as left atrial plication, neochordae implantation, edge-to-edge repair, or a combination of these techniques, with or without associated tricuspid valve repair and/or other cardiac procedures, including coronary artery bypass grafting, aortic valve surgery, ventricular septal defect repair, atrial septal defect repair, and others, and with or without atiral fibrillation procedures including atrial appendage occlusion |
| TEER for AFMR |
| Fulfills above criteria reporting at least one outcome of interest related to AFMR |
| Exclusion criteria |
| Study population with VFMR |
| Non FMR etiologies (rheumatic, degenerative, ischemic) |
| AFMR managed solely with medical treatment |

**AFMR:** atrial functional mitral regurgitation; **TEER:** transcatheter edge-to-edge repair

**Supplementary Table S3:** List of variables extracted for meta-analysis.

| Data type | Variables |
| --- | --- |
| Study | Author name(s), publication year, study period, study center(s), study design |
| Demographics and baseline | Mean age, female percent, hypertension, diabetes, chronic kidney disease, coronary artery disease, chronic obstructive pulmonary disease, dyslipidemia, preoperative data such as NYHA class, ejection fraction, atrial fibrillation, EuroSCORE II and/or STS-PROM scores |
| Preproceural | Left atrial diameter, LVEDD, LVESD, LVEDV, concomitant tricuspid regurgitation (TR >2+), concomitant cardiac procedures |
| Postprocedural outcome | Follow-up duration, reduction in MR severity, early stroke, early acute kidney injury, early all-cause mortality, early cardiac-specific mortality, early HF hospitalization, early reoperation/reintervention, postprocedural atrial and ventricular reverse remodeling parameters (left atrial diameter, LVESD, LVEDD, mean ejection fraction, late reoperation, late all-cause mortality, late cardiac specific mortality, late post-procedural NYHA III/IV, late stroke, late HF hospitalization/readmission |

**NYHA:** New York Heart Association; **EuroSCORE**: European System for Cardiac Operative Risk Evaluation; **STS-PROM Score:** Society of Thoracic Surgeons Predicted Risk of Mortality; **LVEDD:** left ventricular end-diastolic diameter; **LVESD:** left ventricular end-systolic diameter; **LVEDV:** left ventricular end-diastolic volume; **TR:** Tricuspid Regurgitation; **MR:** Mitral Regurgitation; **HF**: heart failure

**Supplementary Table S4.** Newcastle-Ottawa scale (NOS) risk of bias (quality) assessment.

| Study | Arm | Study years | Selection | Comparability | Outcomes | Total (/9 stars) |
| --- | --- | --- | --- | --- | --- | --- |
| Hirji 2020 | S | 2002-2019 | **** | * | *** | 8 |
| Takahashi 2020 | S | 2008-2016 | *** | * | *** | 7 |
| Deferm 2021 | S | 2010-2018 | *** | * | *** | 7 |
| Balogh 2020 | S | 2003-2017 | *** | * | *** | 7 |
| Sakagushi 2019 | S | NA | **** | ** | ** | 8 |
| Morisaki 2022 | S | 2008-2021 | **** | * | *** | 8 |
| Masanao 2020 | S | NA | ** |  | * | 3 |
| Vohra 2012 | S | 2007-2011 | **** | * | *** | 8 |
| Kaneyuki 2020 | S | 2011-2018 | **** | * | *** | 8 |
| Chen 2020 | S | 2008-2018 | *** | * | *** | 7 |
| Matsumori 2020 | S | 2000-2019 | **** | * | *** | 8 |
| Tanaka 2020 | S | 2015-2018 | *** | * | *** | 7 |
| Carino 2021 | S | NA | *** | * | *** | 7 |
| Kim 2023 | S | 2000-2020 | *** | * | *** | 7 |
| Mesi 2021 | S | 2010-2018 | *** | * | *** | 7 |
| Kihara 2009 | S | 2000- 2004 | ** | * | ** | 5 |
| Kawamoto 2022 | S | 2001-2019 | *** | * | *** | 7 |
| Ye 2023 | S | 2010 - 2019 | *** | * | *** | 7 |
| Fan 2023 | S | 2012- 2015 | *** | * | *** | 7 |
| Wagner 2022 | S | 2000-2020 | *** | * | *** | 7 |
| Tanaka 2022 | I (E-E) | 2010-2021 | *** |  | *** | 6 |
| Doldi 2022 | I (E-E) | 2008-2019 | *** | * | *** | 7 |
| Yoon 2022 | I (E-E) | 2007- 2020 | *** | * | *** | 7 |
| Benito‐González 2021 | I (E-E) | 2012-2021 | *** | * | *** | 7 |
| Claeys 2021 | I (E-E) | 2011-2019 | *** | * | *** | 7 |
| Li 2022 | I (E-E) | 6/2021-11/2021 | *** |  | *** | 6 |
| Simard 2022 | I (E-E) | 2014-2020 | *** | * | *** | 7 |
| Sodhi 2022 | I (E-E) | 2018-2019 | *** | * | *** | 7 |
| Masiero 2023 | I (E-E) | 2016-2020 | *** | * | *** | 7 |
| Rottländer 2022 | I (E-E) | 2014-2020 | *** | * | *** | 7 |
| Yoshida 2021 | I (E-E) | 2010-2018 | *** | * | *** | 7 |
| Popolo 2021 | I (E-E) | 2009- 2021 | *** | * | *** | 7 |

**Supplementary Table S5.** Preprocedural Echocardiographic characteristics of the included studies.

| study | Lt atrial diameter (mm), mean (SD) | Lt atrial volume (ml), mean (SD) | Lt atrial volume index (ml/m2), mean (SD) | LVEDD (mm), mean (SD) | LVEDV (ml), mean (SD) | LVESD (mm), mean (SD) | LVESV (ml), mean (SD)/median (IQR) | EROA (cm2) mean (SD) | MR volume (ml), mean (SD) | concomitant >+2 TR % |
| --- | --- | --- | --- | --- | --- | --- | --- | --- | --- | --- |
| Hirji 2020^2^ | 50.5 (13.7) | NA | NA | 49.5 (7.8) | NA | NA | NA | NA | NA | 62.8 |
| Takahashi 2020^3^ | NA | 164 (108.0) | 108 (71) | 54 (8.7) | NA | 35 (6.4) | NA | NA | NA | 68.8 |
| Deferm 2021^4^ | NA | NA | 47 (13.3) | NA | 106 (30.3) | NA | 8 (36–56) | NA | NA | 73.3 |
| Balogh 2020^5^ | 47.16 (7.1) | NA | NA | 49.8 (6.4) | NA | NA | NA | NA | NA | NA |
| Sakagushi 2019^6^ | NA | NA | 93.9 (58,8) | 52.9 (8.2) | NA | 34.6 (5.9) | NA | NA | NA | NA |
| Morisaki 2022^7^ | NA | NA | 131.4 (93.0) | 59.5 (5.7) | NA | NA | NA | NA | NA | 87.5 |
| Ohba Masanao 2020^8^ | NA | NA | NA | NA | NA | NA | NA | NA | NA | 100 |
| Vohra 2012^9^ | 60.1 (10.6) | NA | NA | 56 (7.0) | NA | 40 (7.0) | NA | NA | NA | 70 |
| Kaneyuki 2020^10^ | 60 (11.0) | NA | NA | 55 (7.0) | NA | 37 (7.0) | NA | NA | NA | 78 |
| Chen 2020^11^ | 54.1 (9.0) | NA | NA | 51.8 (5.2) | NA | 34.6 (5.0) | NA | NA | NA | 80.4 |
| Matsumori 2020^12^ | 62.3 (13.5) | NA | NA | 50.1 (6.1) | NA | 32.6 (3.8) | NA | 0.39 (0.09) | 60.7 (16.3) | 86.4 |
| Tanaka 2020^13^ | 50 (15.3) | NA | NA | 56.1 (9.8) | NA | 41 (10.6) | NA | 0.44 (0.18) | 55.4 (11.7) | 80 |
| Carino 2021^14^ | 50 (6.9) | NA | NA | 47.2 (5.6) | NA | NA | NA | NA | NA | 65 |
| Kim 2023^15^ | 55.7 (12.0) | 100.9 (73.5) | NA | NA | 53.6 (10.1) | NA | 36 (9.0) | NA | NA | 69.4 |
| Mesi 2021^16^ | NA | 110 (50.7) | NA | NA | 106 (34.0) | NA | NA | 0.41 (0.09) | NA | 59 |
| Kihara 2009^17^ | NA | NA | NA | NA | 79 (15.0) | NA | 30 (9) | NA | NA | 75 |
| Kawamoto 2022^18^ | 59 (14.0) | 194 (275.0) | NA | 57 (8.5) | 160 (53.3) | 38 (6.8) | 61 (44–85) | NA | NA | 34 |
| Ye 2023^19^ | 51 (3.3) | NA | NA | 43.6 (4.1) | NA | 31 (5.7) | NA | 0.467 (0.3) | NA | NA |
| Fan 2023^20^ | NA | 119 (49.0) | NA | 49 (6) | NA | 37 (6.1) | NA | NA | NA | 76.7 |
| Wagner 2022^21^ | 49 (2.2) | NA | NA | 50 (2.2) | NA | NA | NA | NA | NA | 50.4 |
| Tanaka 2022^22^ | NA | 63.9 (27.4) | NA | 75 (28.8) | NA | 30 (14.0) | NA | 0.344 (0.9) | 55.9 (18.3) | 80.5 |
| Doldi 2022^23^ | NA | 65.7 (34.6) | NA | NA | 53.3 (25.6) | NA | 22.6 (12.8) | 0.27 (0.1) | NA | 63.5 |
| Yoon 2022^24^ | 48.8 (7.5) | 69.7 (39.6) | NA | 48.8 (7.5) | 38.8 (11.9) | 32 (6.7) | 14.8 (5.5) | 0.3 (0.1) | NA | 75.8 |
| Benito‐González 2021^25^ | NA | 106.5 (75.5) | NA | NA | 101.9 (35.0) | NA | 52.2 (27.4) | NA | NA | 56.8 |
| Claeys 2021^26^ | NA | 72 (33.0) | NA | 30 (4.0) | NA | NA | NA | NA | NA | 27 |
| Li 2022^27^ | 83.6 (9.5) | NA | NA | 56.6 (5.4) | NA | 38.8 (6.7) | NA | NA | NA | NA |
| Simard 2022^28^ | NA | 85 (49.0) | NA | 55 (8.0) | NA | 37 (7.0) | NA | NA | NA | 81 |
| Sodhi 2022^29^ | 49 (10.0) | NA | NA | 53 (6.0) | 116.9 (41.2) | 38 (6.0) | 46.6 (17.9) | NA | NA | 23.5 |
| Masiero 2023^30^ | 28 (4.4) | 52 (20.0) | NA | 28 (2.9) | 53 (11.8) | 19 (2.9) | NA | 0.37 (0.09) | NA | 23 |
| Rottländer 2022^31^ | NA | NA | 60.8 (9.1) | 47.9 (1.8) | NA | NA | NA | 0.2 (0.03) | 37.9 (3.3) | 75 |
| Yoshida 2021^32^ | NA | NA | 87 (35.5) | 50 (7.4) | NA | 33 (7.4) | NA | 0.3 (0.1) | 46 (23.7) | 65 |
| Popolo 2021^33^ | 50 (7.4) | 102 (40.0) | 62 (23.7) | 50 (7.4) | 89 (32.5) | 33 (8.1) | 39 [30–50] | 0.34 (0.1) | NA | 47 |

**LVEDD:** Left Ventricular End-Diastolic Diameter; **LVEDV:** Left Ventricular End-Diastolic Volume; **LVESD:** Left Ventricular End-Systolic Diameter; **EROA:** Effective Regurgitant Orifice Area; **MR:** Mitral Regurgitation; **TR:** Tricuspid Regurgitation.

**Supplementary Table S6:** Weighted mean demographic criteria among A) surgery and B) intervention groups

|  | A) Surgery group | B) Intervention |
| --- | --- | --- |
| Variables | (Weighted mean* ± SD) | (Weighted mean* ± SD) |
| Age | 69.52±4.68 | 79.44±1.4 |
| Female percent | 48.32±14.22 | 55.33±4.96 |
| Preprocedural EF | 59.68±15.6 | 57.98±6.74 |
| Preprocedural HF (NYHA class III/IV) | 41.4±12.9 | 81.96±10.66 |
| Hypertension | 53.02±20.7 | 79.08±4.22 |
| Diabetes | 25.53±22.14 | 24.07±5.32 |
| CKD | 15.51±10.03 | 24.07±5.32 |
| CAD | 10.08±10.45 | 44.11±16.29 |
| COPD | 19.29±22.14 | 19.81±6.06 |
| Dyslipidemia | 37.32±17.85 | 36.75±10.32 |
| Smoking | 37.28±8.98 | 9±0 |
| Pre procedural concomiant AF | 89.28±15.85 | 86.33±11.98 |
| Procedural Euro score II mean | 3.87±1.75 | 5.05±1.57 |
| Pre procedural STS risk score mean | 2.48±3.26 | 6.46±1.65 |
| Preprocedural Lt atrial diameter (mm) | 54.07±4.19 | 51.88±9.97 |
| Preprocedural Lt atrial volume (ml) | 137.58±36.48 | 77.1±17.27 |
| Preprocedural Lt atrial volume index (ml/m^2^) | 95.08±33.22 | 69.93±11.29 |
| LVEDD (mm) | 52.1±4.35 | 49.43±15.34 |
| LVEDV (ml) | 100.92±32.85 | 75.48±29.12 |
| LVESD (mm) | 36.08±3.02 | 32.6±4.94 |
| Preprocedural EROA (cm^2^)¶ | 0.43±0.02 | 0.3±0.04 |
| Preprocedural MR volume ml mean | 58.05±2.41 | 46.6±5.57 |
| Pre procedural concomitant ≥ 2+ TR | 71.58±12.5 | 56.19±17.22 |

¶ **denotes regurgitation severity**

**EF:** Ejection Fraction; **HF:** Heart Failure; **NYHA:** New York Heart Association; **CKD:** Chronic Kidney Disease; **CAD:** Coronary Artery Disease; **COPD:** Chronic Obstructive Pulmonary Disease; **AF:** Atrial Fibrillation; **Euro SCORE**: European System for Cardiac Operative Risk Evaluation; **STS Score:** Society of Thoracic Surgeons Risk Score; **LVEDD:** Left Ventricular End-Diastolic Diameter; **LVEDV:** Left Ventricular End-Diastolic Volume; **LVESD:** Left Ventricular End-Systolic Diameter; **EROA:** Effective Regurgitant Orifice Area; **MR:** Mitral Regurgitation; **TR:** Tricuspid Regurgitation.

**Supplementary Table S7:** numbers and % of AF ablation and TR procedures among the surgical group vs. TEER group.

|  | **No. (%)** |  | **No. (%)** |
| --- | --- | --- | --- |
| **Among surgical group** |  | **Among transcatheter group** |  |
| Associated tricuspid valve procedure | 746/1165 (64.0%) |  | Zero |
| Associated AF procedure | 863/1162 (74.3%) |  | Zero |
| **Among surgical group with AF** |  | **Among transcatheter group with AF** |  |
| Associated AF procedure | 863/1073 (80.4%) |  | Zero |

**Supplementary Table S8:** details of AF ablation and TR procedures among the surgical group.

| Study/year | **Surgery group** | **Surgery group** |
| --- | --- | --- |
| **Study/year** | **Tricuspid valve procedures (No, and %)** | **Atrial fibrillation ablation procedures (No, and %)** |
| Hirji 2022 | **36** (38.3%) | **20 (**21.3%) MAZE |
| Takahashi 2020 | **45** (100%) | **8** (18%) MAZE, **30 (**67%) LAA exclusion |
| Deferm 2021 | **53** (54.7%) | **29** (29.9%) MAZE, **40** (41.2%) LAA closure |
| Balogh 2020 | **68** (49%) | **97** (70%) MAZE, LAA exclusion |
| Sakagushi 2019 | **16** (80%) | **20** (100%) LAA exclusion, **13** (65%) MAZE |
| Morisaki 2022 | **31** (100%) in both PA and MVR groups | **0** (0%, in both groups) MAZE, **25** (**11** in PA, and **14** in MVR) LAA exclusion |
| Masanao 2020 | **3** (100%) | NR |
| Vohra 2012 | **12** (60%) | **20** (100%) (LAA exclusion, MAZE, and pulmonary vein isolation) |
| Kaneyuki 2020 | **31** (78%) | **20** (50%) MAZE |
| Chen 2020. | **72** (87.8%) | **52** (63.4%) (MAZE, LAA exclusion) |
| Matsumori 2020 | **19** (86.4%), TVr **17** (77.3%), **TVR** 2 (9.1%) | **9** (40.9%) MAZE |
| Tanaka 2020 | **8** (80%) | **6** (100%) (2 (20%) MAZE, **4** (40%) pulmonary vein isolation) |
| Carino 2021 | **14** (70%) | **14** (70%) MAZE |
| Kim 2023 | **25** (69.4%), TVr **24** (66.7%), **TVR** 1 (2.8) | **36** (100%) (**36** (100%) MAZE, **20** (55.6%) LAA exclusion |
| Mesi 2021 | **15** (38.5%) | **11** (28.2%) (**5**(12.8%) MAZE, **6** (15.4%) LAA exclusion) |
| Kihara 2009 | **5** (41.7%) | **12** (100%) pulmonary vein isolation |
| Kawamoto 2022 | **41**(82%) (**40** (80%) TVr annuloplasty, **1** (2%) TVR) | **50** (100%) , **20** (40%) **MAZE,** **33** (66%) LAA exclusion |
| Qing Ye 2023 | **144** (58.3%) (TVr annuloplasty) | **247** (100%) MAZE |
| Xingli Fan 2023 | **46** (76.7%) (TVr annuloplasty) | **60** (100%) MAZE |
| Wagner M 2022 | **62** (50.4%) (TVr annuloplasty) | **74** (60.2%) concomitant MAZE and LAA exclusion |

**LAA:** Left Atrial Appendage; **TVr**: Tricuspid Valve Repair; **TVR:** Tricuspid Valve Replacement.

**Supplementary Fig. S1:** PRISMA flow diagram of our included studies

**Identification of studies via databases and registers**

Records removed *before screening*:

Duplicate records removed (n =1639)

Records marked as ineligible by automation tools (n =0)

Records removed for other reasons (n =0)

Records identified from*:

Ovid MEDLINE (n=1835)

Ovid Embase (n=3578)

Cochrane Library (n=103)

**Identification**

Records screened.

(n = 3877)

Records excluded**

(n = 3750)

Reports sought for retrieval.

(n = 127)

Reports not retrieved.

(n = 4)

**Screening**

Reports assessed for eligibility.

(n =123)

Reports excluded: 91.

1-Not fulfilling the inclusion criteria or no outcomes for AFMR (n =58)

2-Abstracts, letters (n =7)

3-Review article (n =25)

5-Studies including overlapped patients, in the same period, the same hospital, and the same authors (n=1)

Studies included in review.

(n = 32)

**Included**

**Supplementary Figure 2:** Funnel plots and leave-one-out analysis for the late severe MR (+3, and +4): A) Entire group, B) Surgery group, and C) intervention.

| ***I)Late severe MR*** | Funnel plot | Leave-one-out |
| --- | --- | --- |
| ***Entire group*** | 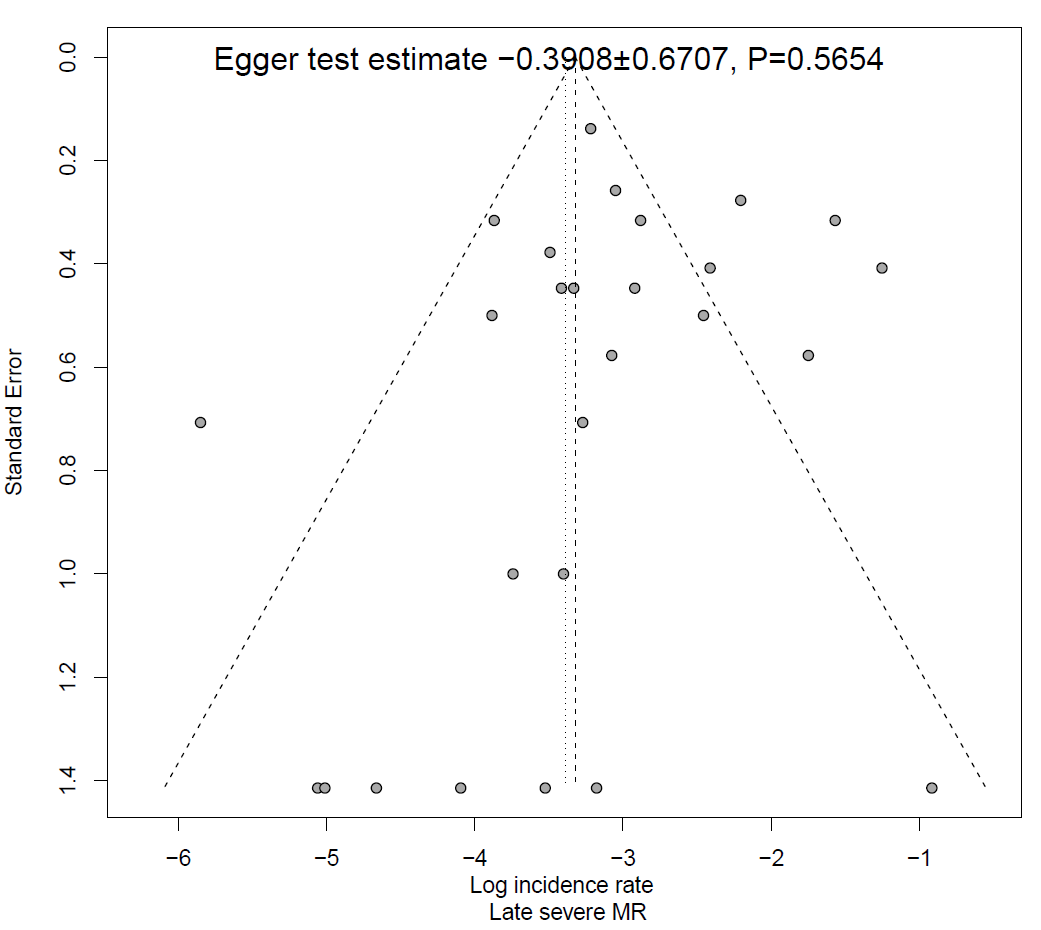 | 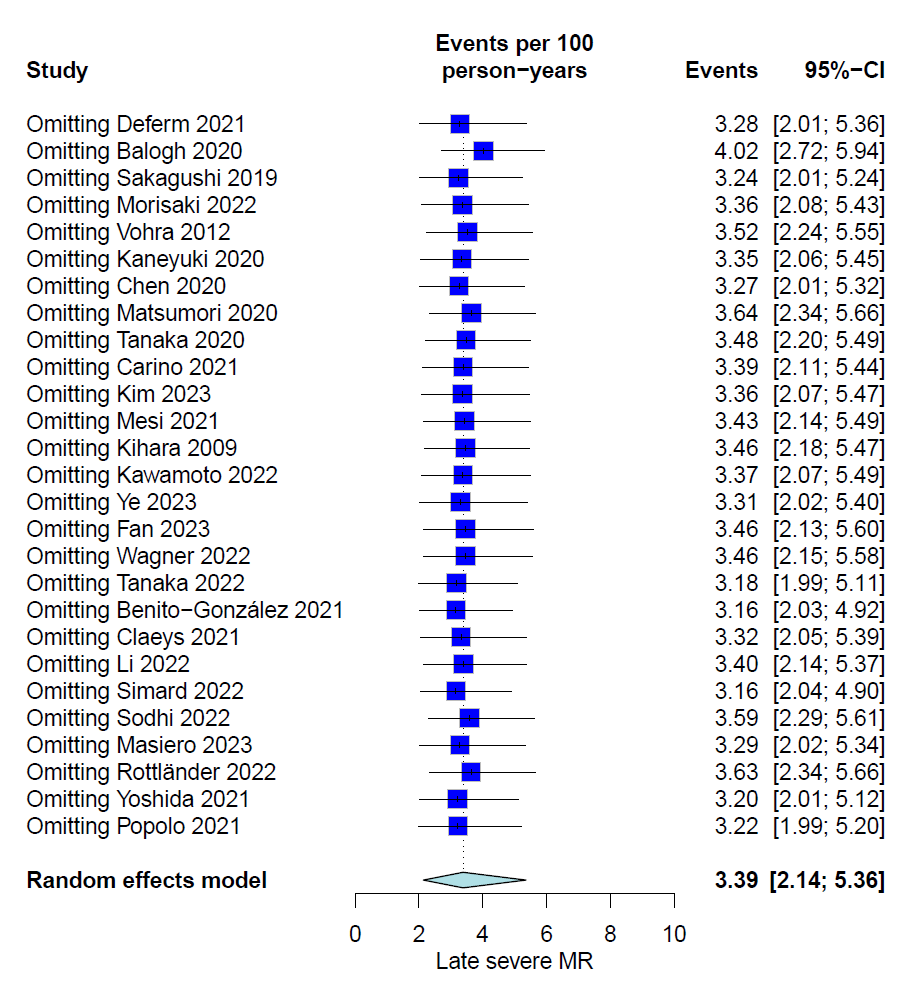 |
| **Surgery** | 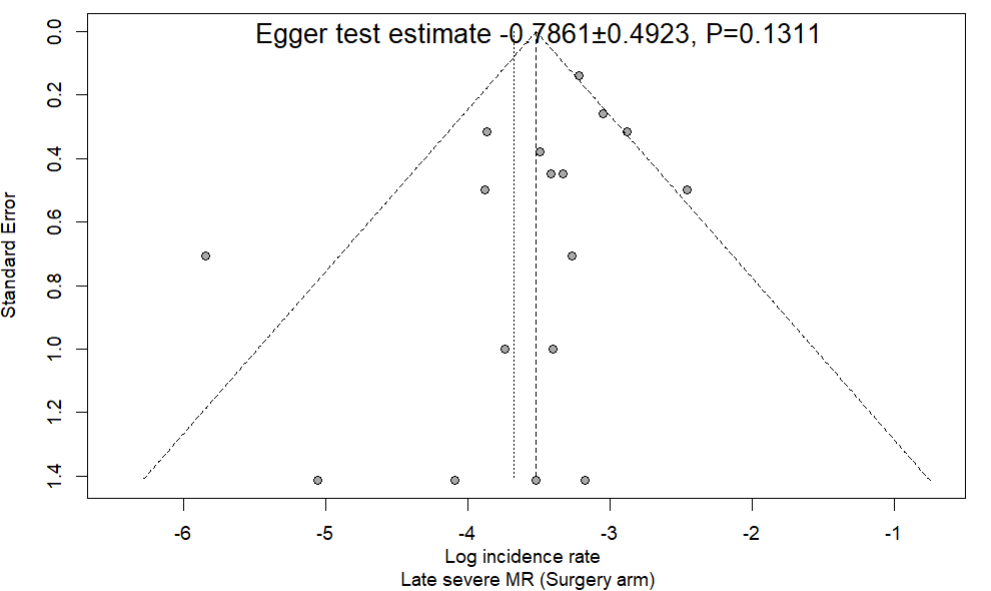 | 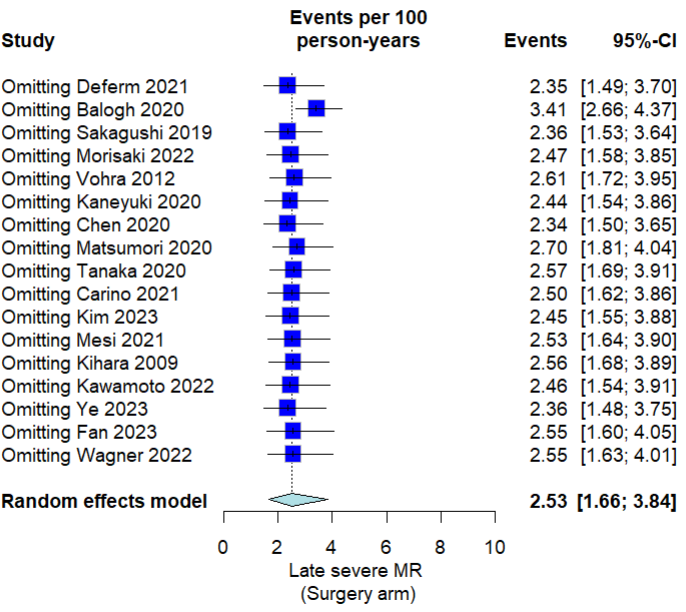 |
| **Interventional** | 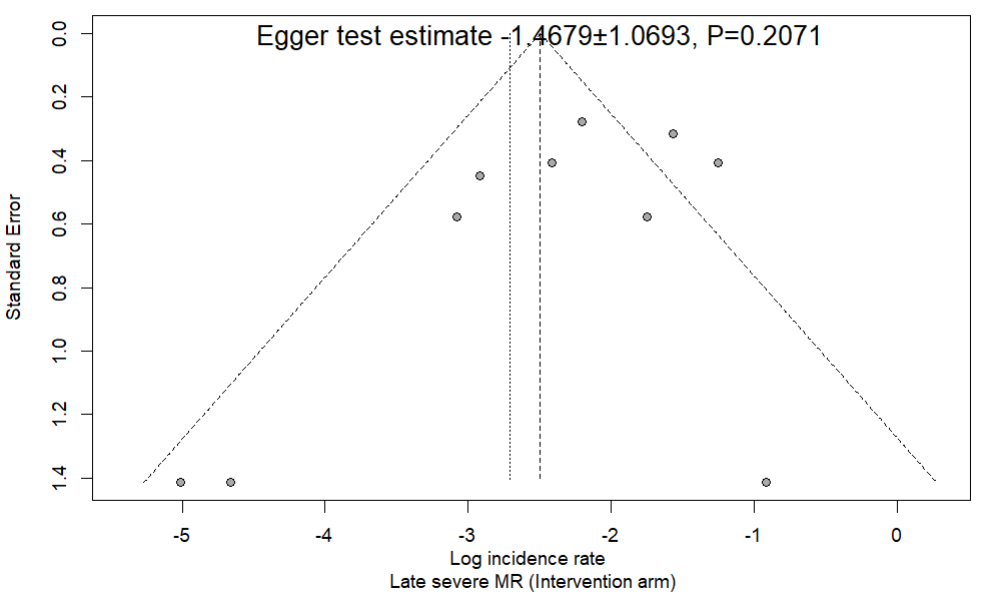 | 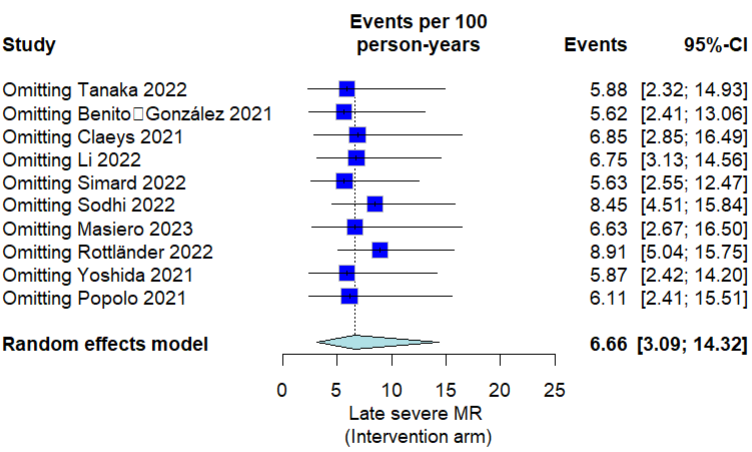 |

**Supplementary Fig. S3.** Forest plot of subgroup differences between surgery group and TEER group for the pooled events of Early acute kidney injury (AKI).


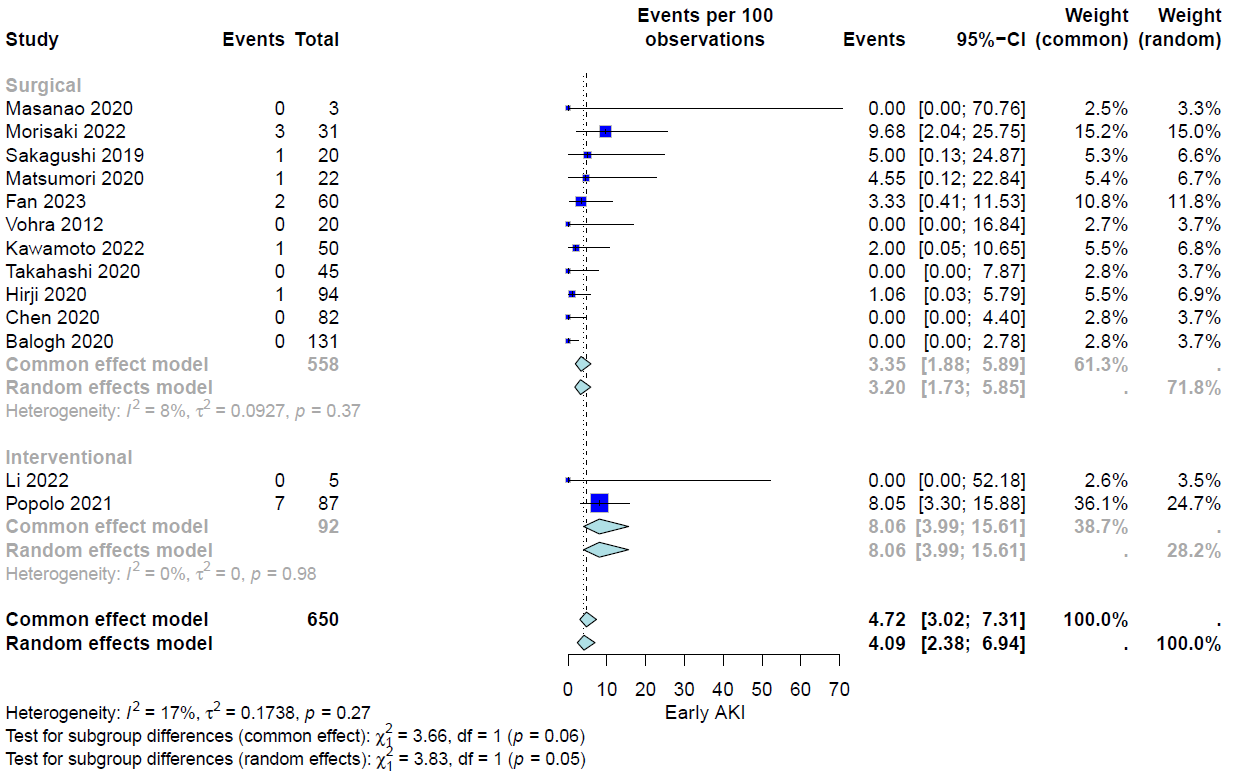


**Supplementary Fig. S4.** Forest plot of subgroup differences between surgery group and TEER group for the pooled events of Early all-cause mortality.


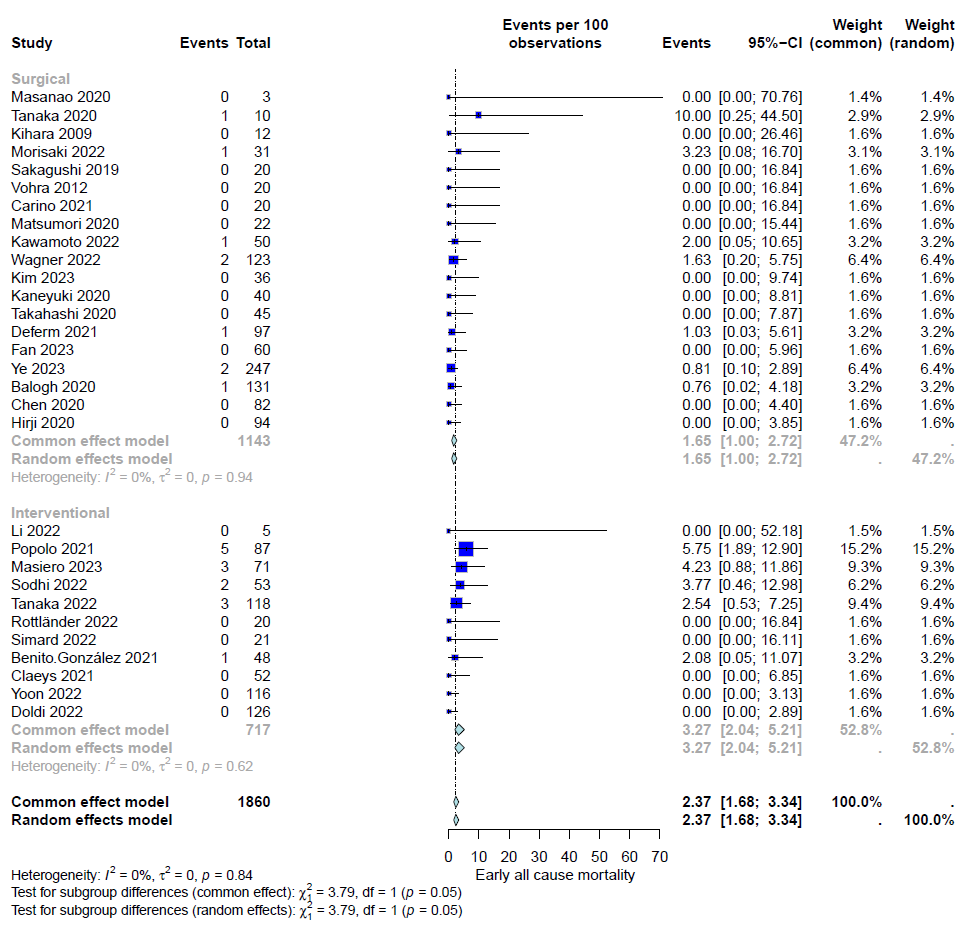


**Supplementary Fig. S5.** Forest plot of subgroup differences between surgery group and TEER group for the pooled events of Early cardiac-specific mortality.


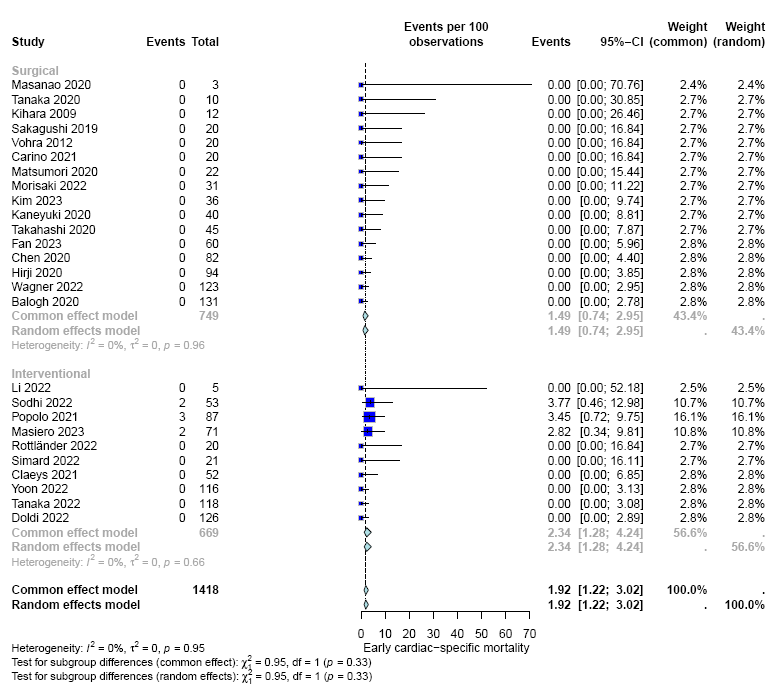


**Supplementary Fig. S6.** Forest plot of subgroup differences between surgery group and TEER group for the pooled events of Early stroke.


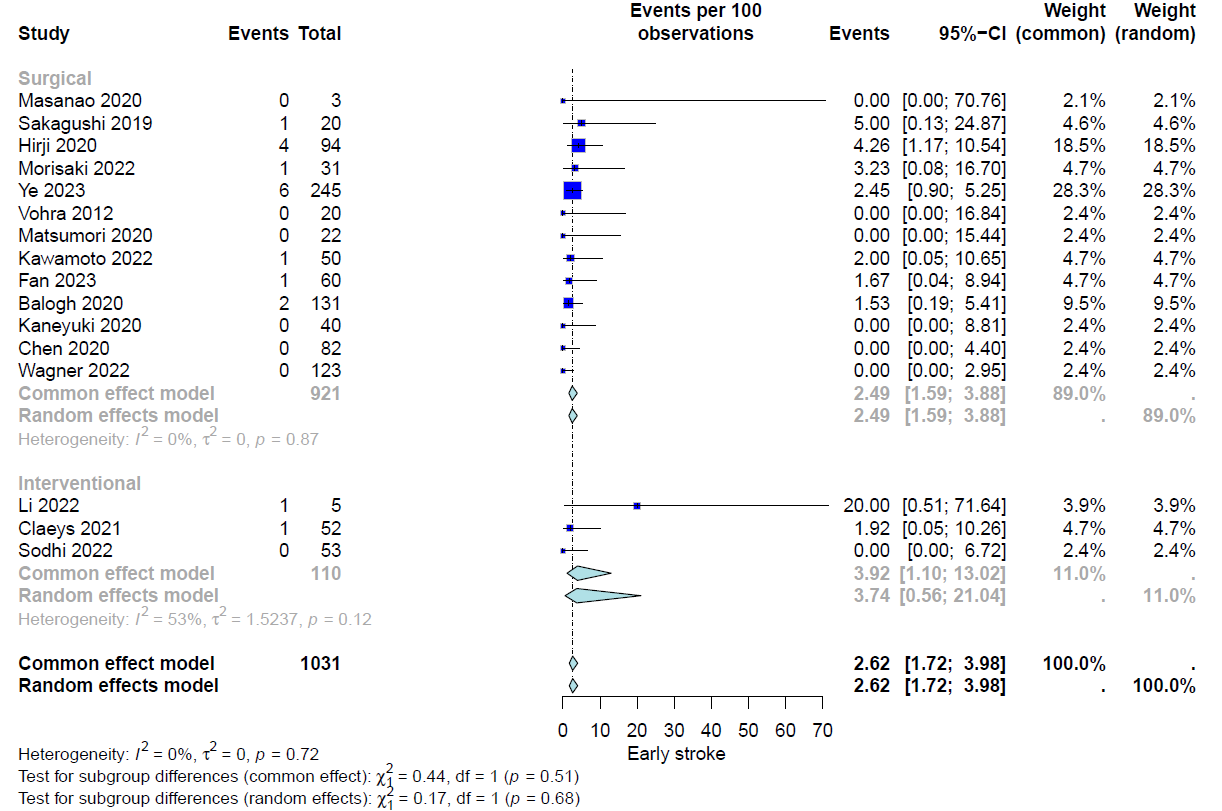


**Supplementary Fig. S7.** Forest plot of subgroup differences between surgery group and TEER group for the pooled events of Early heart failure (HR) hospitalization/re-admission.


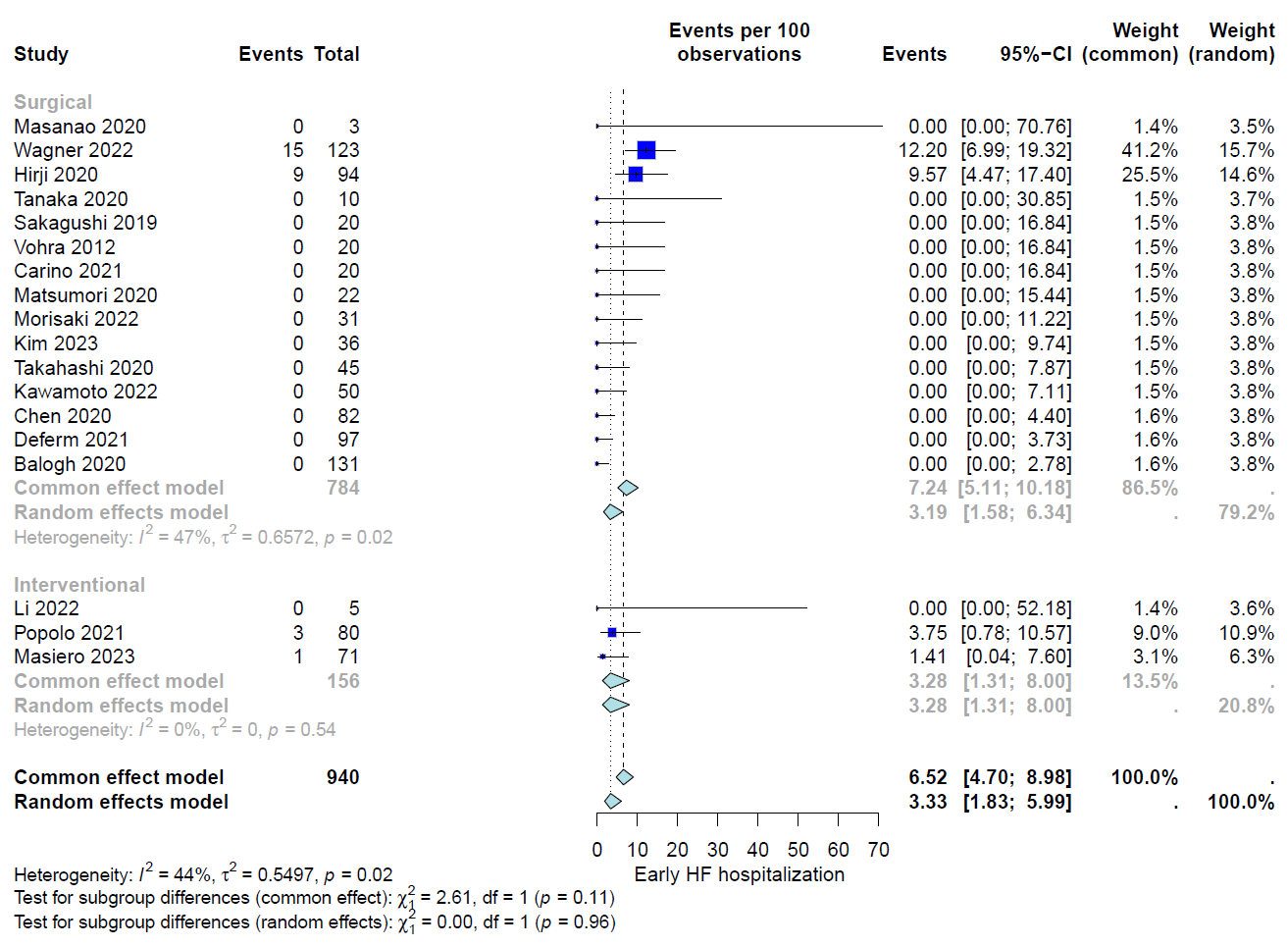


**Supplementary Fig. S8.** Forest plot of subgroup differences between surgery group and TEER group for the pooled events of Early reoperation/reintervention.


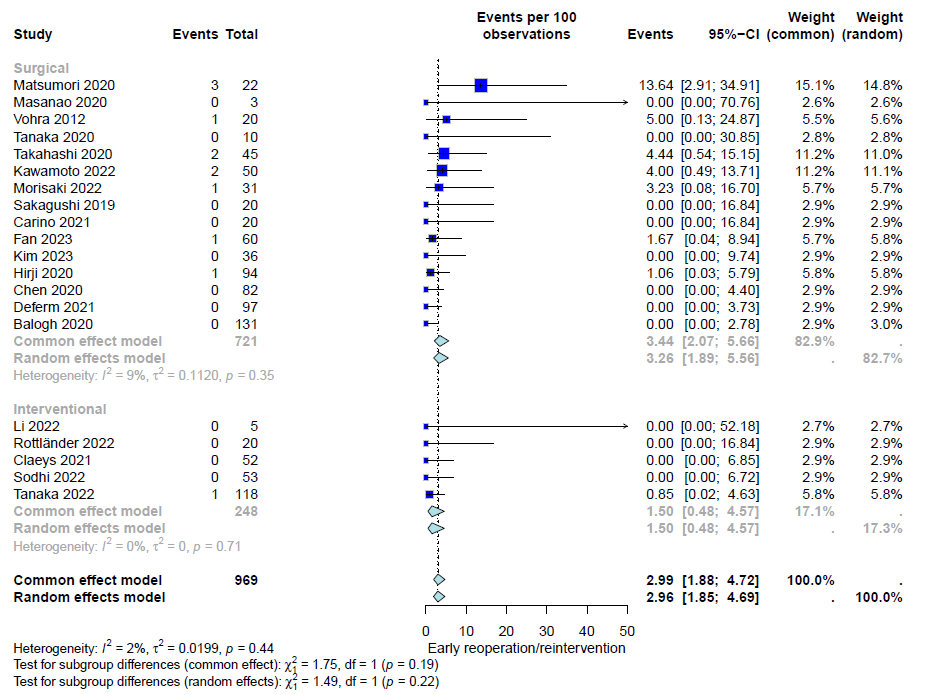


**Supplementary Fig. S9.** Forest plot of subgroup differences between surgery group and TEER group for postprocedural mean Lt atrial diameter (mm).


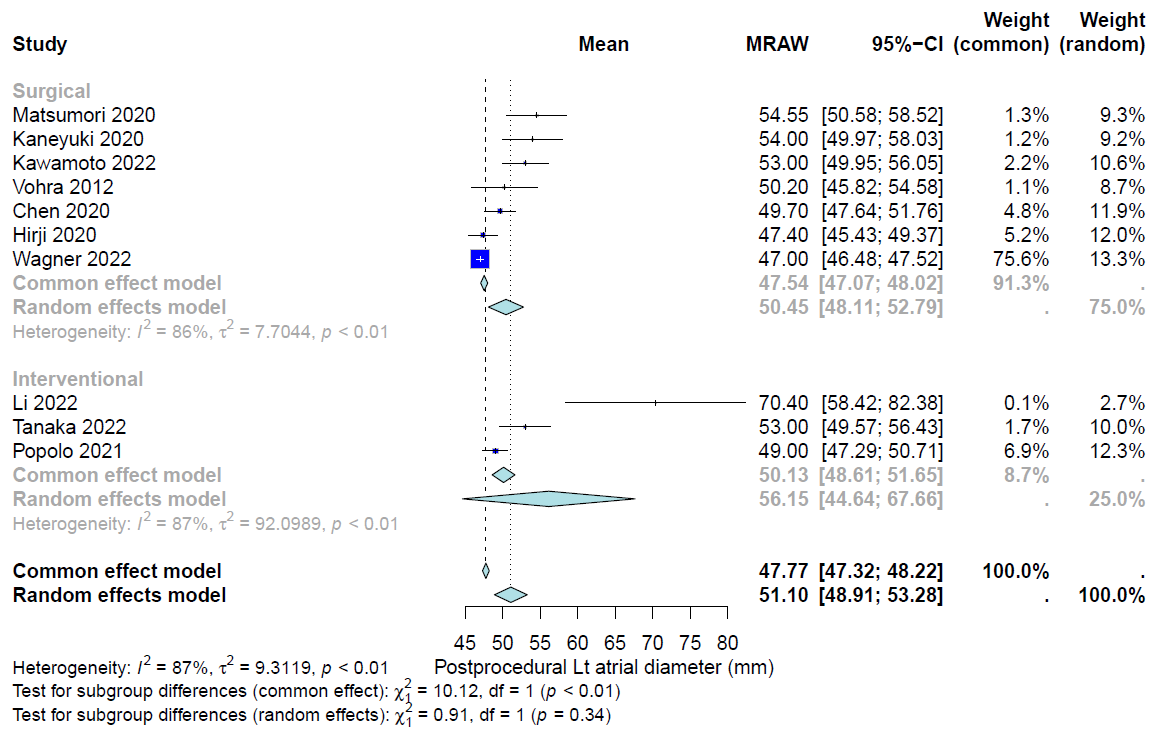


.

**Supplementary Fig. S10.** Forest plot of subgroup differences between surgery group and TEER group for post-procedural mean left ventricular end-systolic diameter LVESD (mm).


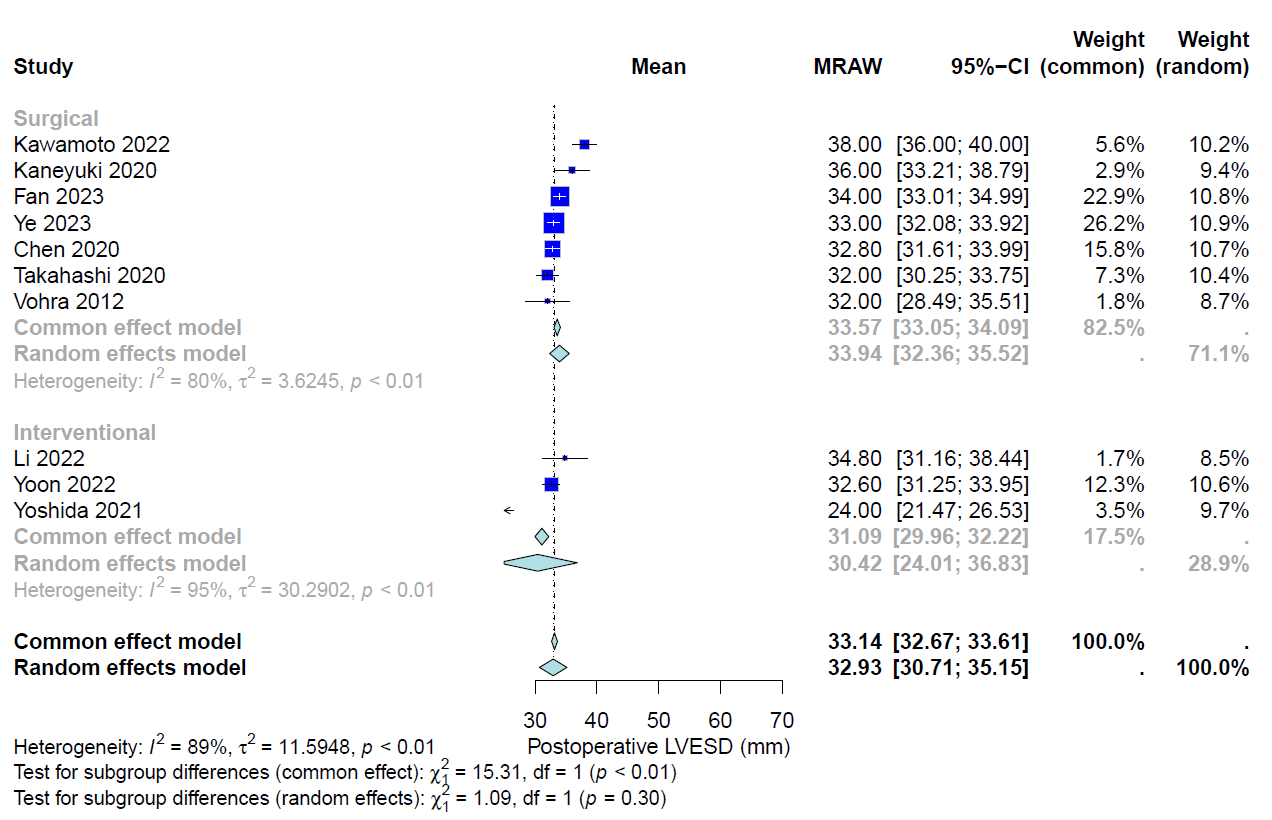


**Supplementary Fig. S11.** Forest plot of subgroup differences between surgery group and TEER group for post-procedural mean left ventricular end-systolic diameter LVEDD (mm).


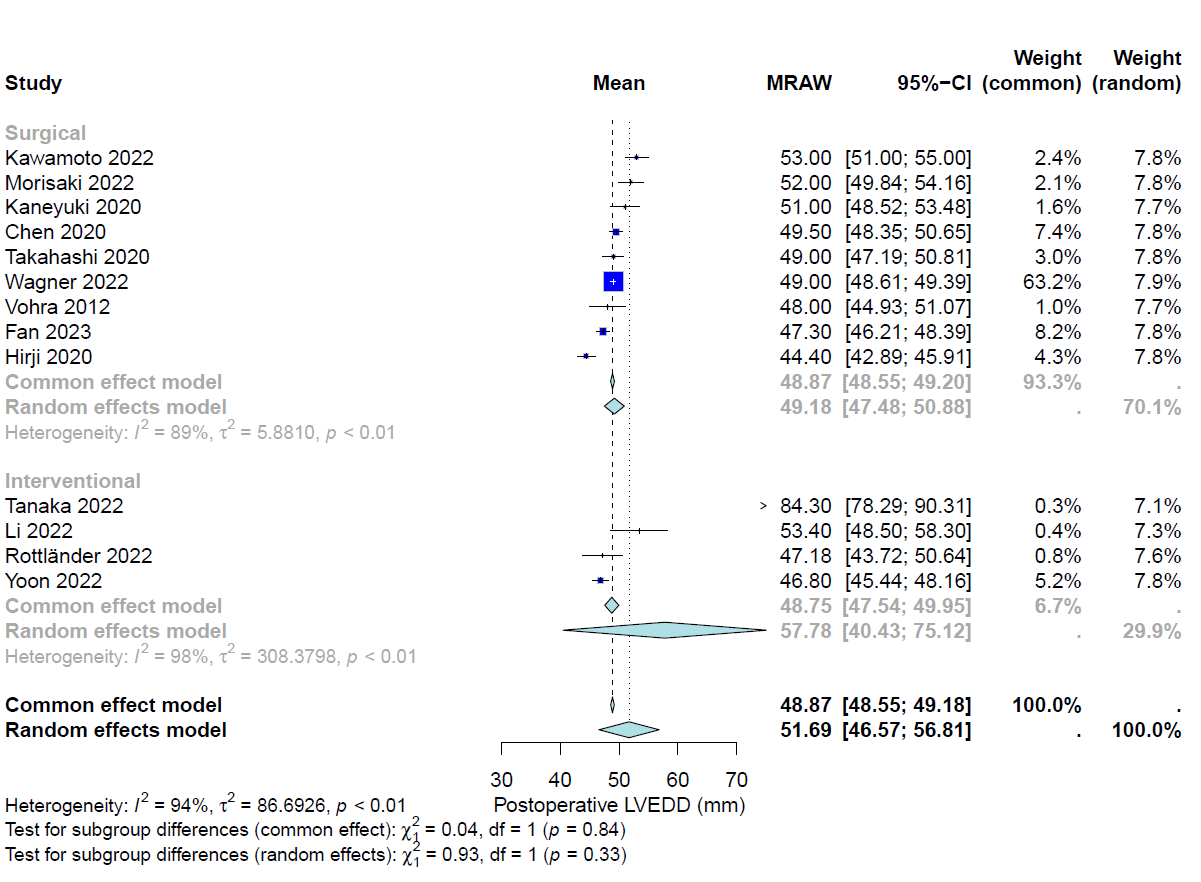


**Supplementary Fig. S12.** Forest plot of subgroup differences between surgery group and TEER group for post-procedural mean ejection fraction (EF).


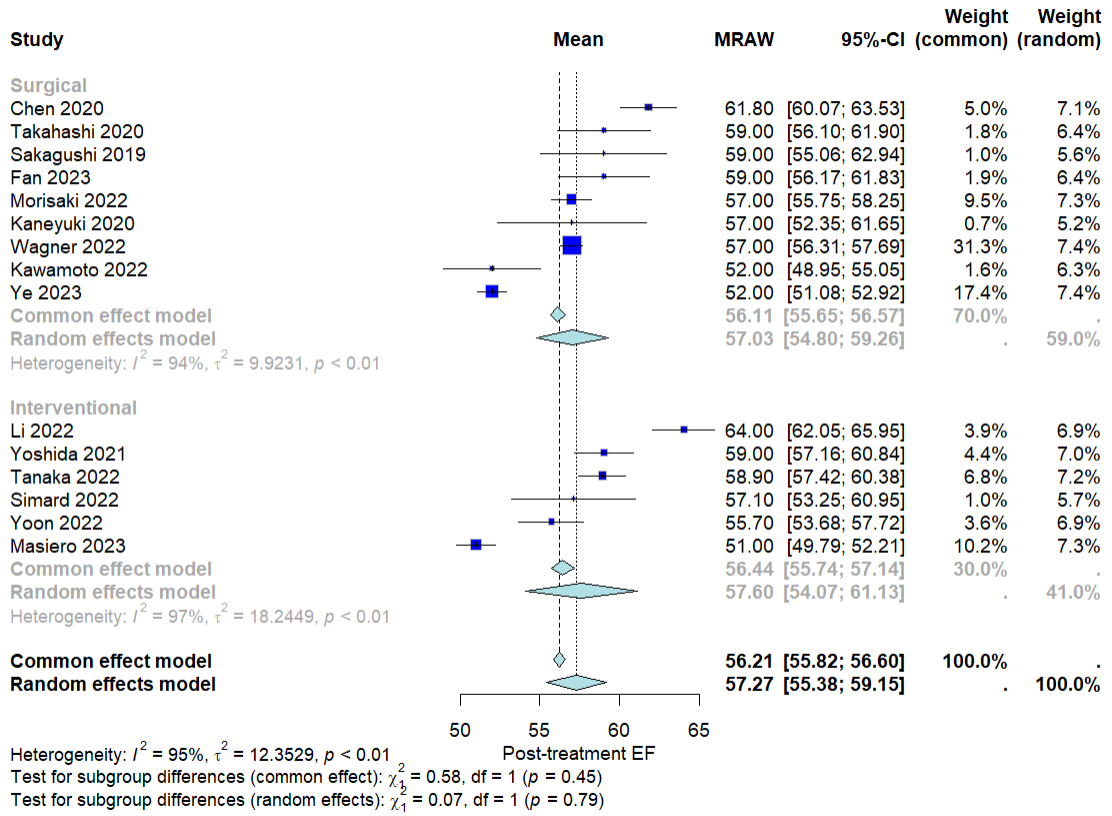


**Supplementary Fig. S13.** Forest plot of subgroup differences between surgery group and TEER group for the incidence rate (IR) of late reoperation.


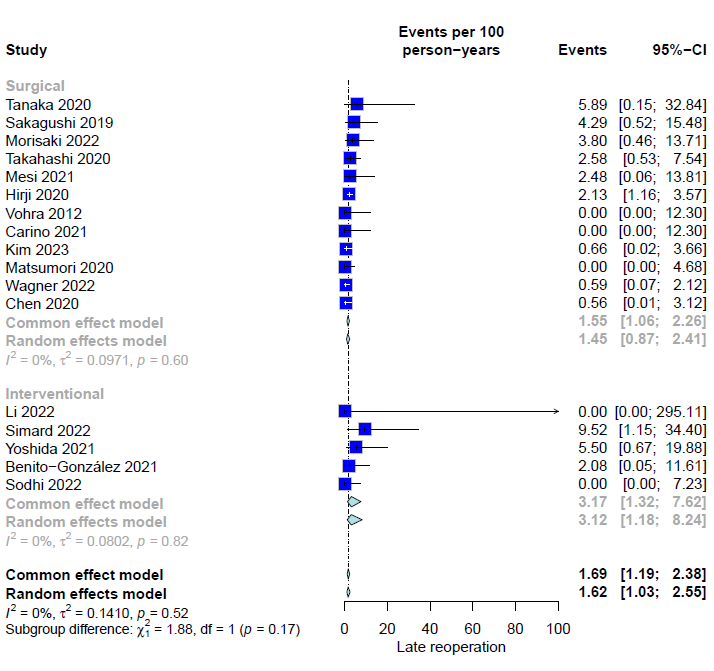


**Supplementary Fig. S14.** Forest plot of subgroup differences between surgery group and TEER group for the incidence rate (IR) of late (follow up) cardiac-specific mortality.


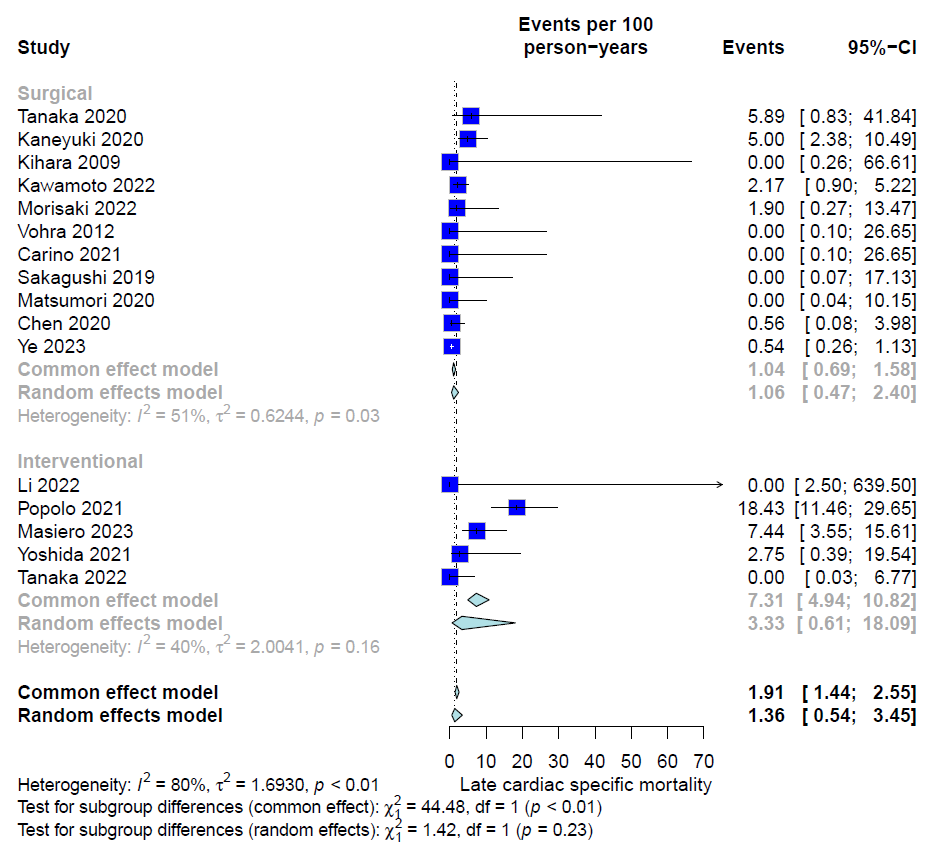


**Supplementary Fig. S15.** Forest plot of subgroup differences between surgery group and TEER group for the incidence rate (IR) of late (follow up) stroke.


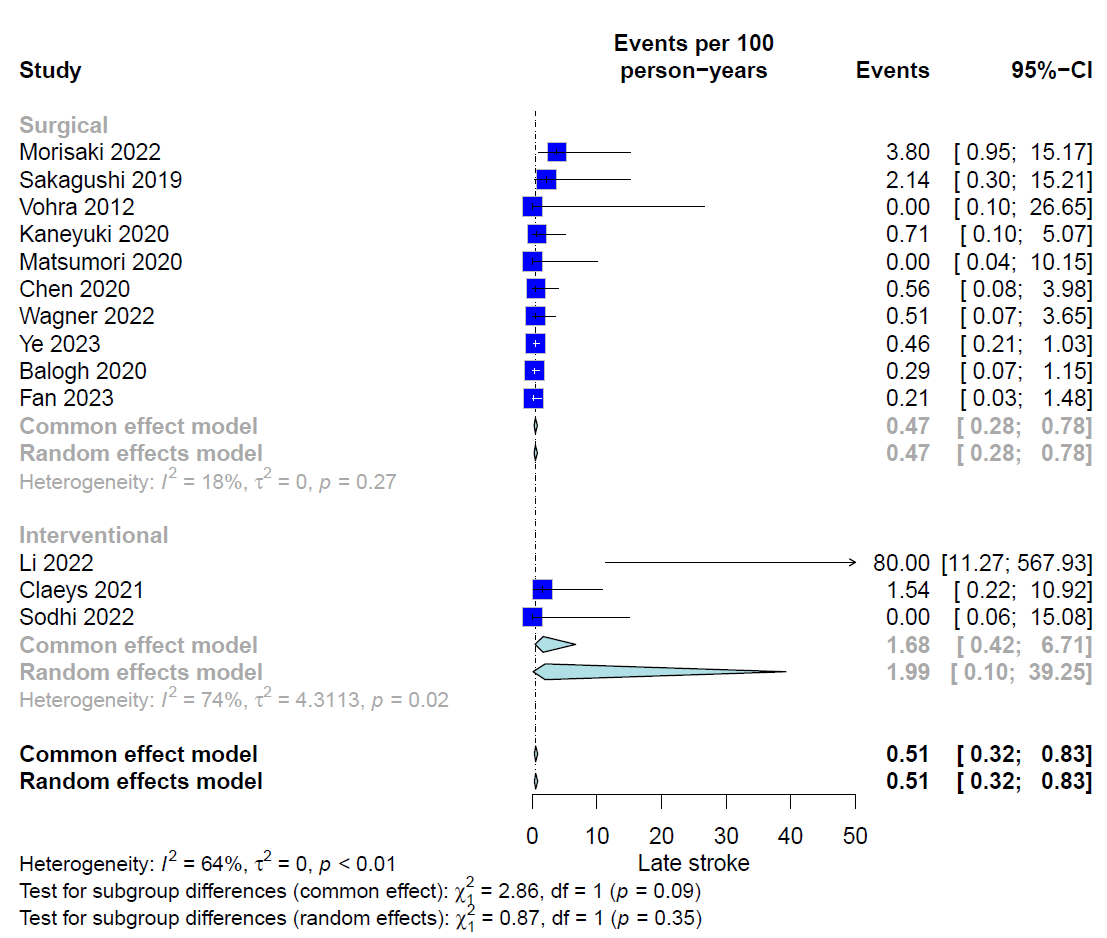


1. Amabile A, Fereydooni S, Mori M, et al. Variable definitions and treatment approaches for atrial functional mitral regurgitation: A scoping review of the literature. *Journal of Cardiac Surgery*. 2022;37(5):1182-1191.

2. Hirji SA, Cote CL, Javadikasgari H, Malarczyk A, McGurk S, Kaneko T. Atrial functional versus ventricular functional mitral regurgitation: prognostic implications. *The Journal of Thoracic and Cardiovascular Surgery*. 2022;164(6):1808-1815. e4.

3. Takahashi Y, Abe Y, Takashi M, et al. Mid-term results of valve repairs for atrial functional mitral and tricuspid regurgitations. *General Thoracic and Cardiovascular Surgery*. 2020;68:467-476.

4. Deferm S, Bertrand PB, Verhaert D, et al. Outcome and durability of mitral valve annuloplasty in atrial secondary mitral regurgitation. *Heart*. 2021;107(18):1503-1509.

5. Balogh Z, Mizukami T, Bartunek J, et al. Mitral valve repair of atrial functional mitral regurgitation in heart failure with preserved ejection fraction. *Journal of Clinical Medicine*. 2020;9(11):3432.

6. Sakaguchi T, Totsugawa T, Orihashi K, et al. Mitral annuloplasty for atrial functional mitral regurgitation in patients with chronic atrial fibrillation. *Journal of Cardiac Surgery*. 2019;34(9):767-773.

7. Morisaki A, Takahashi Y, Fujii H, Sakon Y, Murakami T, Shibata T. Patch augmentation vs. valve replacement for patients with atrial functional mitral regurgitation and long-standing atrial fibrillation. *Journal of Thoracic Disease*. 2022;14(10):3831.

8. Ohba M AS, Kabasawa M, Ito T, Matsuo K, Hayashida N, et al. Surgical Repair for Atrial Functional Mitral Regurgitation. *American Journal of Surgery Case Reports*. 2020;1(1):1002.

9. Vohra HA, Whistance RN, Magan A, Sadeque SA, Livesey SA. Mitral valve repair for severe mitral regurgitation secondary to lone atrial fibrillation. *European journal of cardio-thoracic surgery*. 2012;42(4):634-637.

10. Kaneyuki D, Nakajima H, Asakura T, et al. Midterm results of mitral valve repair for atrial functional mitral regurgitation: a retrospective study. *Journal of Cardiothoracic Surgery*. 2020;15:1-7.

11. Chen J, Wang Y, Lv M, et al. Mitral valve repair and surgical ablation for atrial functional mitral regurgitation. *Annals of Translational Medicine*. 2020;8(21)

12. Matsumori M, Kawashima M, Aihara T, et al. Efficacy of left atrial plication for atrial functional mitral regurgitation. *General Thoracic and Cardiovascular Surgery*. 2021;69:458-465.

13. Tanaka C, Akasaka K, Ushioda R, et al. Examination of anterior leaflet pseudoprolapse causing severe mitral regurgitation and its ideal surgical procedure. 2020:E205-E211.

14. Carino D, Lapenna E, Ascione G, et al. Is mitral annuloplasty an effective treatment for severe atrial functional mitral regurgitation? *Journal of Cardiac Surgery*. 2021;36(2):596-602.

15. Kim K, Kim HJ, Jung S-H, Lee J, Kim JB. Functional insufficiency of mitral and tricuspid valves associated with atrial fibrillation: impact of postoperative atrial fibrillation recurrence on surgical outcomes. *Korean Circulation Journal*. 2023;53(8):550-562.

16. Mesi O, Gad MM, Crane AD, et al. Severe atrial functional mitral regurgitation: clinical and echocardiographic characteristics, management and outcomes. *Cardiovascular Imaging*. 2021;14(4):797-808.

17. Kihara T, Gillinov AM, Takasaki K, et al. Mitral regurgitation associated with mitral annular dilation in patients with lone atrial fibrillation: an echocardiographic study. *Echocardiography*. 2009;26(8):885-889.

18. Kawamoto N, Fukushima S, Kainuma S, et al. Mitral valve surgery for atrial functional mitral regurgitation: predicting recurrent mitral regurgitation and mid-term outcome. *General Thoracic and Cardiovascular Surgery*. 2022;70(9):761-769.

19. Ye Q, Zhao Y, Bai C, et al. Outcome of mitral repair combined with Cox-maze procedure for atrial functional mitral regurgitation with heart failure with recovered ejection fraction. *European Journal of Cardio-Thoracic Surgery*. 2023;64(2):ezad273.

20. Fan X, Tang Y, Ma Y, et al. Mitral valve repair and concomitant maze procedure versus catheter ablation in the treatment of atrial functional mitral regurgitation. *BMC Cardiovascular Disorders*. 2022;22(1):543.

21. Wagner CM, Brescia AA, Watt TM, et al. Surgical strategy and outcomes for atrial functional mitral regurgitation: All functional mitral regurgitation is not the same! *The Journal of Thoracic and Cardiovascular Surgery*. 2022;

22. Tanaka T, Sugiura A, Öztürk C, et al. Transcatheter edge-to-edge repair for atrial secondary mitral regurgitation. *Cardiovascular Interventions*. 2022;15(17):1731-1740.

23. Doldi P, Stolz L, Orban M, et al. Transcatheter mitral valve repair in patients with atrial functional mitral regurgitation. *Cardiovascular Imaging*. 2022;15(11):1843-1851.

24. Yoon S-H, Makar M, Kar S, et al. Outcomes after transcatheter edge-to-edge mitral valve repair according to mitral regurgitation etiology and cardiac remodeling. *Cardiovascular Interventions*. 2022;15(17):1711-1722.

25. Benito-González T, Carrasco-Chinchilla F, Estévez-Loureiro R, et al. Clinical and echocardiographic outcomes of transcatheter mitral valve repair in atrial functional mitral regurgitation. *International Journal of Cardiology*. 2021;345:29-35.

26. Claeys MJ, Debonnaire P, Bracke V, et al. Clinical and hemodynamic effects of percutaneous edge-to-edge mitral valve repair in atrial versus ventricular functional mitral regurgitation. *The American journal of cardiology*. 2021;161:70-75.

27. Li W, Long Y, Pan W, et al. Transcatheter edge to edge repair using the ease-of-use valve clamp system for functional mitral regurgitation: a primary report. *Surgery Today*. 2023;53(1):90-97.

28. Simard T, Reddy YN, Thaden JJ, et al. Atrial mitral regurgitation: Characteristics and outcomes of transcatheter mitral valve edge‐to‐edge repair. *Catheterization and Cardiovascular Interventions*. 2022;100(1):133-142.

29. Sodhi N, Asch FM, Ruf T, et al. Clinical outcomes with transcatheter edge-to-edge repair in atrial functional MR from the EXPAND study. *Cardiovascular Interventions*. 2022;15(17):1723-1730.

30. Masiero G, Montonati C, Rubbio AP, et al. Impact of Transcatheter Edge-to-Edge Mitral Valve Repair on Atrial Functional Mitral Regurgitation from the GIOTTO Registry. *The American Journal of Cardiology*. 2024;211:219-227.

31. Rottlaender D, Golabkesh M, Degen H, Oeguetcue A, Saal M, Haude M. Mitral valve edge‐to‐edge repair versus indirect mitral valve annuloplasty in atrial functional mitral regurgitation. *Catheterization and Cardiovascular Interventions*. 2022;99(6):1839-1847.

32. Yoshida J, Ikenaga H, Nagaura T, et al. Impact of percutaneous edge-to-edge repair in patients with atrial functional mitral regurgitation. *Circulation Journal*. 2021;85(7):1001-1010.

33. Rubbio AP, Testa L, Grasso C, et al. Transcatheter edge-to-edge mitral valve repair in atrial functional mitral regurgitation: insights from the multi-center MITRA-TUNE registry. *International Journal of Cardiology*. 2022;349:39-45.
